# Differential effect of plakoglobin in restoring the tumor suppressor activities of p53-R273H vs. p53-R175H mutants

**DOI:** 10.1371/journal.pone.0306705

**Published:** 2024-10-03

**Authors:** Chu Shiun Lo, Parnian Alavi, Blessing Bassey-Archibong, Nadia Jahroudi, Manijeh Pasdar

**Affiliations:** 1 Department of Oncology, Faculty of Medicine and Dentistry, University of Alberta, Edmonton, Alberta, Canada; 2 Department of Medicine, Faculty of Medicine and Dentistry, University of Alberta, Edmonton, Alberta, Canada; 3 Department of Biology and Environmental Sciences Concordia University of Edmonton, Edmonton, Alberta, Canada; Virginia Commonwealth University, UNITED STATES OF AMERICA

## Abstract

The six most common missense mutations in the DNA binding domain of p53 are known as “hot spots” and include two of the most frequently occurring p53 mutations (p53-R175H and p53-R273H). p53 stability and function are regulated by various post-translational modifications such as phosphorylation, acetylation, sumoylation, methylation, and interactions with other proteins including plakoglobin. Previously, using various carcinoma cell lines we showed that plakoglobin interacted with wild-type and several endogenous p53 mutants (e.g., R280K, R273H, S241F, S215R, R175H) and restored their tumor suppressor activities *in vitro*. Since mutant p53 function is both mutant-specific and cell context-dependent, we sought herein, to determine if plakoglobin tumor suppressive effects on exogenously expressed p53-R273H and p53-R175H mutants are similarly maintained under the same genetic background using the p53-null and plakoglobin-deficient H1299 cell line. Functional assays were performed to assess colony formation, migration, and invasion while immunoblotting and qPCR were used to examine the subcellular distribution and expression of specific proteins and genes that are typically regulated by or regulate p53 function and are altered in mutant p53-expressing cell lines and tumors. We show that though, plakoglobin interacted with both p53-R273H and p53-R175H mutants, it had a differential effect on the transcription and subcellular distribution of their gene targets and their overall oncogenic properties *in vitro*. Notably, we found that plakoglobin’s tumor suppressive effects were significantly stronger in p53-R175H expressing cells compared to p53-R273H cells. Together, our results indicate that exploring plakoglobin interactions with p53-R175H may be useful for the development of cancer therapeutics focused on the restoration of p53 function.

## Introduction

*TP53* encodes a transcription factor and tumor suppressor protein (p53) that plays essential roles in the maintenance of genome integrity, cell cycle progression, DNA damage repair and apoptosis and other physiological processes such as metabolism and immune response [1–3]. It is therefore not surprising that p53 mutations occur in 50% of cancers and is associated with tumor aggressiveness and resistance to conventional cancer therapeutics [4, 5]. p53 mutants often lose the tumor suppressor function of the wild-type p53 protein (p53-WT), and/or gain new oncogenic functions (gain-of-function; GOF) that activate oncogenic gene expression and pathways thereby promoting tumorigenesis [6–16]. The majority of p53 mutations are missense and in the DNA binding domain (DBD) [17, 18] with the six most frequently occurring DBD mutations (R175, Y220, G245, R248, R273, or R282) [3] accounting for ~30% of p53 mutations and referred to as the “hot spots” [6, 17, 19]. Mutants can be classified into two categories: conformational/structural and contact [3, 17, 20–23]. While both categories are compromised in their ability to bind to p53-WT target DNA sites, conformational mutants (R175H, G245S, R249S, and R282H) do so by disrupting the conformation and folding of the p53 protein whereas contact mutants (R273H, R248W, and R248Q) do so by affecting the amino acid residues that are directly involved in p53-WT DNA binding while maintaining a p53-WT-like structure [3, 17, 20–23].

We recently demonstrated that the cell adhesion protein plakoglobin interacts with endogenously expressed WT as well as various p53 mutants (e.g., R280K, R273H, S241F, S215R, R175H) endogenously expressed in cells of different tissue origins and genetic backgrounds [24–28]. Of the p53 mutants that plakoglobin has been shown to interact with, two (the conformational p53-R175H and contact p53-R273H mutants) comprise the most frequently occurring p53 mutants that have been reported to function in a cell-context dependent manner [29–34].

In this study, we sought to determine the effects of plakoglobin on p53-R273H and p53-R175H mutants expressed in the same genetic background. The value of this investigation is to clarify and confirm plakoglobin’s tumorigenic effect on the oncogenic functions of p53-R273H and p53-R175H mutants without the confounding contribution of cellular context. Since there are no currently available cancer cell lines that endogenously express all three proteins (plakoglobin, p53-R273H and p53-R175H), we used the p53-null and plakoglobin-deficient H1299 non-small cell lung carcinoma cell line [25] for our studies and exogenously expressed plakoglobin, p53-R273H and p53-R175H at respective intervals in the H1299 cells to address our research question.

H1299 cells have been used routinely to assess the function of exogenously expressed WT and mutant p53 proteins under the same genetic background and to the best of our knowledge, H1299 is the only available plakoglobin-deficient and p53 null cell line that is uniquely suited for this study. We show for the first time that plakoglobin differentially affected the oncogenic properties of p53-R273H and p53-R175H mutants expressed in the same cell line–H1299. Specifically, we found that plakoglobin co-expression led to a stronger inhibition of the tumorigenic properties of the conformational p53-R175H mutant relative to that of the contact p53-R273H mutant. We propose that plakoglobin interaction with p53-R175H mutant and its subsequent tumorigenic functions can be exploited for the development of effective therapies for p53-R175H-expressing tumors focused on the restoration of wild-type p53 functions.

## Materials and methods

### Cell lines and culture conditions

All tissue culture reagents were purchased from Gibco unless otherwise stated. The human plakoglobin deficient and p53 null non-small cell lung carcinoma cell line H1299 was provided by Dr. Roger Leng, University of Alberta and has been previously described (**S1 Fig**) [25]. H1299 cells and its transfectants were maintained in minimum essential medium (MEM) supplemented with 10% fetal bovine serum (HyClone Laboratories, USA), 1% penicillin-streptomycin and 5 μg/mL kanamycin (complete MEM, CMEM). SKOV-3 ovarian carcinoma cell line was purchased from American Type Culture Collection (ATCC) and cultured in Dulbecco’s modified eagle medium: F12 medium (DMEM/F12, 1:1) supplemented with 10% fetal bovine serum, 1% penicillin-streptomycin and 5 μg/mL kanamycin.

### Plasmid construction and transfection

HA-tagged p53, pcDNA3-plakoglobin-FLAG and p53^R175H^ (p53 in which arginine 175 is replaced with histidine) expression constructs and their respective H1299 transfectants have been described previously [25, 26, 35, 36]. The pCMV-Neo-Bam-p53^R273H^ (p53 in which arginine 273 is replaced with histidine) plasmid was purchased from Addgene (USA). p53^R273H^ transfectants were generated using the jetOPTIMUS^®^ (Polyplus, France) DNA transfection reagent following the manufacturer’s protocol. Cells were transfected with either 5 μg of pCMV-p53^R273H^ (hereafter H1299-p53-273) alone or with 10 μg of pcDNA3-plakoglobin plasmids (H1299-PG-p53-273). Stable cell lines were selected by supplementing CMEM with 800 μg/mL G418 (p53-273 transfectants) or 800 μg/mL G418 and 600 μg/mL hygromycin (PG-p53-273 transfectants) 48 hours post-transfection. Stable transfectants were maintained in 400 μg/mL G418 (H1299-p53-273) or 400 μg/mL G418 and 400 μg/mL hygromycin (H1299-PG-p53-R273H). p53 and plakoglobin expression were verified using immunofluorescence (not shown) and immunoblot assays. All experiments were carried out with stable transfectants unless indicated otherwise.

### Cell extraction and immunoblotting

Equal amounts of total cellular proteins from 100 mm cultures were processed for extraction with RIPA lysis buffer (150 mM NaCl, 50 mM Tris-HCl pH 7.4, 1% NP-40, 0.25% sodium deoxycholate, 1 mM EDTA, 1 mM PMSF, 1 mM NaF, 1 mM Na_3_VO_4_, and Roche protease inhibitor cocktail, Sigma, Canada) and immunoblotting using primary and secondary antibodies. Antibodies were diluted in 5% gelatin TBST (Tris-buffered saline, 0.1% Tween 20) to their respective working concentrations **(Table 1)** and used for immunoprecipitation (IP) or immunoblot (IB) assays [25, 26, 28]. Blots were scanned and protein bands were quantitated using the NIH ImageJ software. Protein levels were normalized to internal controls (tubulin/actin or lamin) in the same cell line.

**Table 1:** List of primary and secondary antibodies used in various assays.

Antibody	Species	Assays			Manufacturer/Catalogue #
Primary		IP	IB	IF	
AKT2	Mouse	-	1:1000	-	Santa Cruz # sc-5270
pAKT	Rabbit		1:1000	-	Cell Signaling # 9271
β-Actin	Mouse	-	1:1000	-	Santa Cruz # sc-47778
β-catenin	Mouse	-	1:1000	-	MilliporeSigma # c-7207
β-tubulin	Mouse	-	1:1000	-	Developmental Studies Hybridoma Bank (E7)
GST (Glutathione S-transferase)	Goat	-	1:1000	-	Cytiva # 27-4577-01
Lamin B	Rabbit	-	1:1000	-	Abcam # ab16048
Nucleophosmin (NPM)	Mouse	-	1:2000	-	Abcam # ab10530
p53	Mouse	1:100	1:1000	-	Santa Cruz # sc-126
Plakoglobin	Mouse	1:100	1:1000	1:100	BD Bioscience # 610253
**Secondary**					
AlexaFluor 790	Mouse	-	1:30,000	-	Jackson Immuno Research # 211-652-171
AlexaFluor 790	Goat	-	1:30,000	-	Jackson Immuno Research # 115-655-174
AlexaFluor 680	Goat	-	1:30,000	-	Jackson Immuno Research # 115-625-174
Li-Cor IRDye®-800CW	Donkey	-	1:20,000	-	LiCor # LIC926-32214
Alexa 488 anti-mouse IgG	Goat	-	-	1:1000	Molecular Probes/A11029

### Immunoprecipitation

Equal amounts of RIPA extracted total cellular proteins were processed for co-immunoprecipitations with p53 and plakoglobin antibodies or pre-immune serum followed by immunoblotting as described previously [24–26].

### Subcellular fractionation

Cell pellets from 100 mm cultures were extracted with NE-PER™ Nuclear and Cytoplasmic Extraction Reagents (Thermo Scientific) according to the manufacturer’s protocol. Unless otherwise stated, cytoplasmic and nuclear extracts of equal cell numbers were resolved by SDS-PAGE and processed for immunoblot [24, 26]. The purities of cytoplasmic and nuclear extracts were verified by immunoblotting with tubulin and nuclear lamin antibodies.

### Immunofluorescence

SKOV-3 cells were grown to confluency on glass coverslips and processed for staining as described previously [25]. Briefly, coverslips were fixed in ice-cold methanol-acetone (1:1) and processed for indirect immunofluorescence with plakoglobin antibodies followed by secondary antibodies. Antibodies were diluted in PBS-BSA to the concentrations indicated in Table 1. Nuclei were stained with DAPI (1:2000 in PBS) and coverslips were viewed using a Zeiss confocal microscope.

### Preparation and purification of GST-plakoglobin

A construct encoding pGEX-TEV-PG was kindly provided by Dr. William Weis [37]. *Escherichia coli* DH5α cells were transformed by pGEX-TEV-PG constructs to express PG-GST. Cells were grown in Luria-Bertani broth at 37°C to an *A*_600_ of 0.6–0.8 and then induced with 0.5 mM IPTG (isopropyl-β-D-1-thiogalactopyranoside, Thermo Fisher, Canada). Cultures were grown for an additional 6 hours at 30°C, harvested by centrifugation at 4,000 x g at 4°C for 12 min and the supernatants were discarded. Pellets were resuspended in 6 mL of cold bacterial lysis buffer (500 mM NaCl, 0.5% NP-40, 50 mM Tris-HCl pH 7.6, 5 mM EDTA, 5 mM EGTA, 1 mg/mL lysozyme, 10 mM DTT, 2.5 U/mL DNase, 1 mM PMSF and an EDTA-free protease inhibitor cocktail tablet) and lysed via sonication. Lysates were centrifuged at 10,000 x g for 25 min at 4°C and the supernatants were divided into 1 mL aliquots, snap frozen and stored at -80°C.

### GST pull-down assay

A one mL aliquot of PG-GST bacterial lysate was incubated with 150 μL of glutathione sepharose beads (GE Life Sciences) on a rocker-rotator at 4°C for 6 hours. Beads were centrifuged at 21,000 x g for 1 min and supernatants were aspirated. Beads were washed 3x using cold KCl-PBS (0.137 M NaCl, 0.0027 M KCl, 0.01 M Na_2_HPO4, 0.0018 M KH_2_PO_4_) and stored in KCl-PBS at 4°C. Cells from 100 mm cultures of H1299 and H1299-p53-(WT, R175H, R273H) transfectants were extracted in 750 μL RIPA lysis buffer for 20 min on a rocker-rotator at 4°C. The lysates were centrifuged at 48,000 x g for 10 min at 4°C and supernatants were removed. For pull down assays, 600 μL of cell lysates were mixed with 50 μL PG-GST or GST (control) beads and incubated on a rocker rotator for 4–6 hours at 4°C. The beads were washed 3x with RIPA lysis buffer, eluted with 4x SDS sample buffer and processed for immunoblot using p53 and plakoglobin antibodies.

### RNA isolation, RT-PCR and real-time PCR

Cells grown in 100 mm culture dishes were processed for total RNA extraction using the RNeasy Plus Mini Kit (Qiagen) and 1 μg of RNA was used to synthesize cDNA with a QuantiTect Reverse Transcription Kit (Qiagen). For real-time PCR, SYBR Green Master Mix (Thermo Fisher) and specific primers [Integrated DNA Technologies (IDT)] for *PUMA*, *S100A4*, *BAX* and *ACTB* **(Table 2)** were used. Quantitative reverse transcriptase PCR (qRT-PCR) was done using a 7500 ABI Thermocycler. Data were normalized to the internal control (ACTB) and fold changes were calculated based on the 2^−ΔΔCT^ method. Results are presented as means ± SD in histograms constructed by normalizing the values of the H1299 mutant p53 cells to p53-WT cells, and the values of the PG-p53 transfectants to their corresponding p53-only expressing cells.

**Table 2:** List of primers and their sequences used in real-time quantitative PCR assays.

Gene	Primers	Sequences (5-´3´)
*PUMA*	Forward	ACGACCTCAACGCACAGTACGA
	Reverse	CCTAATTGGGCTCCATCTCGGG
*S100A4*	Forward	CAGAACTAAAGGAGCTGCTGACC
	Reverse	CTTGGAAGTCCACCTCGTTGTC
*BAX*	Forward	TCAGGATGCGTCCACCAAGAAG
	Reverse	TGTGTCCACGGCGGCAATCATC
*ACTB*	Forward	CACCATTGGCAATGAGCGGTTC
	Reverse	AGGTCTTTGCGGATGTCCACGT

### *In vitro* soft agar colony formation assay

Cultures were seeded at 7,500 cells in 35 mm culture plates containing 0.6% base agar and 0.35% top agar (Noble Agar, Thermo Scientific) in CMEM. Plates were incubated at 37°C, 5% CO_2_ for two weeks and supplemented with 0.3 mL CMEM every three days. The colonies were fixed and stained with a solution containing 0.05% w/v crystal violet, 1% formaldehyde and 1% methanol in PBS for 20 minutes, destained in water overnight at room temperature and colonies counted under a dissecting microscope. Colony formation results are presented in histograms as means ± SD after normalizing the values of the H1299 mutant p53 cells to p53-WT cells, and the values of the PG-p53 transfectants to their corresponding p53 only expressing cells.

### *In vitro* migration and invasion assays

Migration and invasion assays were performed in triplicates using transwell inserts (Fisher Scientific) as described in detail previously [25, 27, 28]. The number of migrated/invaded cells was counted in five random fields for each membrane using NIH ImageJ Cell Counter software. Migration and invasion results are presented as means ± SD in histograms constructed by normalizing the values of the H1299 mutant p53 cells to p53-WT cells, and the values of the PG-p53 transfectants to their corresponding p53 only expressing cells.

### Statistical data analysis

All quantitative data were presented as mean ± standard deviation (SD). Statistical significance between groups were assessed using Student’s t-test for all assays except qPCR, which was analyzed by One-way ANOVA. p-values < 0.05 were considered significant in all cases. All biochemical experiments were repeated at least 3 times and the figures are representative of one typical experiment for each assay. All functional assays were repeated at least three times and the histograms represent the average of all assays.

## Results

### Plakoglobin interacts with p53-WT, p53-R175H and p53-R273H mutants in H1299 cells

**Fig 1:**
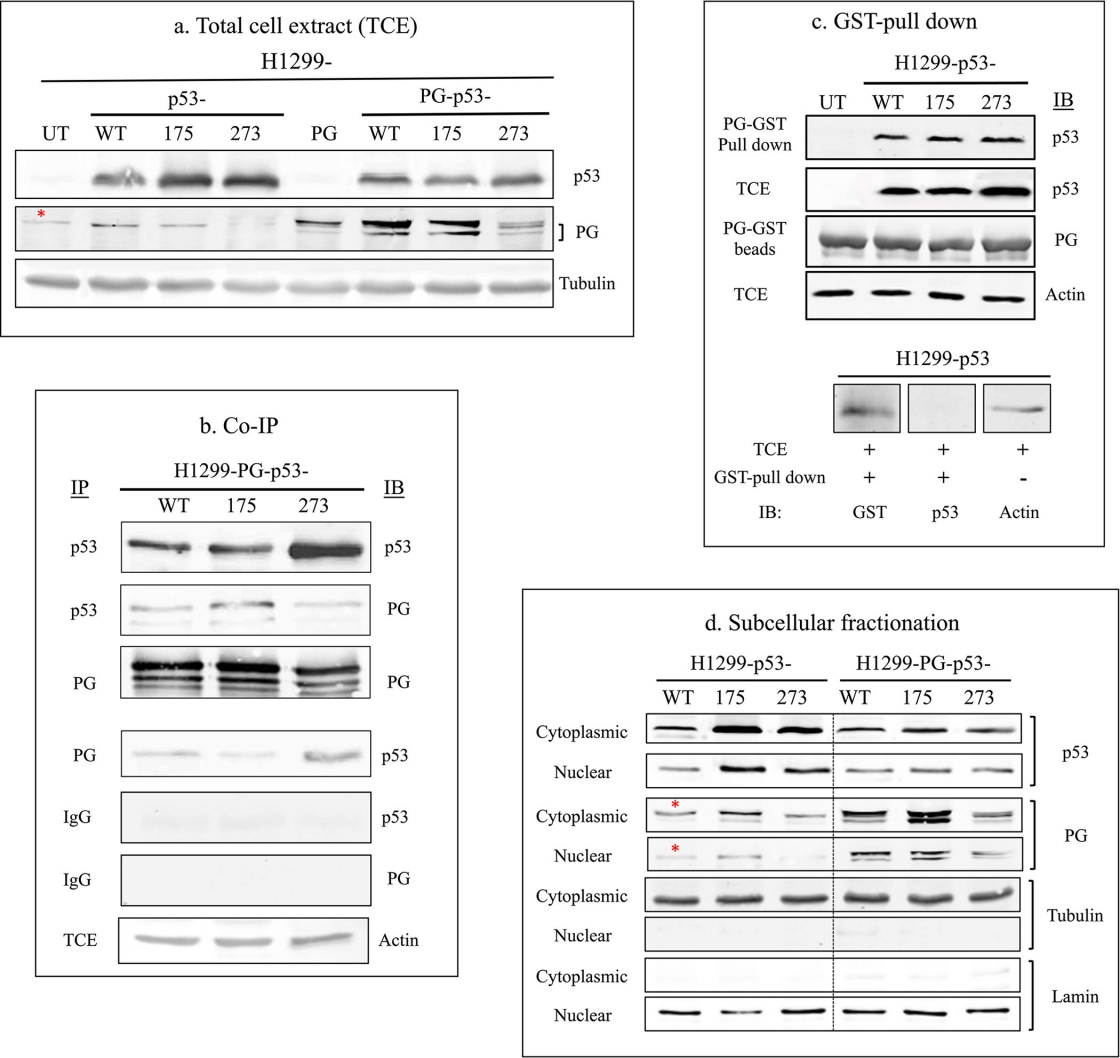
Protein expression and interaction of plakoglobin with p53-WT, p53-R175H conformational and p53-R273H contact mutants. **(a)** Total cellular extracts (**TCE**s) from untransfected H1299 cells (**UT**) and H1299 transfectants expressing p53- (WT, 175, 273) alone or together with plakoglobin (**PG**) [H1299-PG-p53- (WT, 175, 273)] were processed for immunoblot using p53 and plakoglobin antibodies. Beta-tubulin was used as a loading control. **(b)** TCEs from H1299-PG-p53-(WT, 175, 273) were processed for sequential reciprocal co-immunoprecipitation (**Co-IP**) and immunoblotting (**IB**) using p53, plakoglobin and control (IgG) antibodies. Beta-actin was used as loading control. **(c)** Top: TCEs from H1299-p53- (WT, 175, 273) transfectants were incubated with GST-tagged plakoglobin (**PG-GST**) beads and processed for GST-pull down assays and immunoblotting with p53 and plakoglobin antibodies. Samples from the same TCEs ran on a different gel were processed for blotting with actin antibodies. Bottom: TCE from H1299-p53-WT cells was incubated with GST only beads and processed for pull down and immunoblot with GST and p53 antibodies. **(d)** H1299 transfectants were processed for **subcellular fractionation** using NE-PER reagent and cytoplasmic and nuclear extracts of equal cell numbers were processed for immunoblot with p53 and plakoglobin antibodies. The purity of cytoplasmic and nuclear fractions was confirmed by immunoblotting of the equal amount of the same lysates on different gels with tubulin and lamin antibodies respectively, which also served as loading controls. H1299- p53 and H1299-PG-p53 were run on the same gel separated by a lane. * Non-specific band.

As a first step in our investigations, we confirmed that exogenously expressed plakoglobin interacted with exogenously expressed p53-WT, p53-R175H (p53-175) and p53-R273H (p53-273) similarly in H1299 cells. To this end, H1299 cells were transfected with p53 (WT, 175 or 273) with or without plakoglobin. The expression of transfected proteins was validated by immunoblotting of total cell extracts (TCEs) using anti-p53 or -plakoglobin (PG) antibodies **(Fig 1A)**. As mentioned earlier, H1299 cells lack plakoglobin expression [25]. However, we observed a faint non-specific band in plakoglobin immunoblots (depicted with the asterisk * in **Fig 1**) with the specific plakoglobin antibody used in this study. To validate the absence of plakoglobin in H1299 parental cells, we performed immunofluorescence analysis with the same plakoglobin antibody referenced above. As seen in **S1 Fig**, no plakoglobin fluorescent staining was observed in H1299 parental cells **(S1A and S1B Fig)**, which confirmed the absence of plakoglobin in these cells and the non-specific nature of the band observed in our immunoblots.

H1229 cells transiently co-expressing p53- (WT, 175 or 273) and plakoglobin were then processed for sequential and reciprocal co-immunoprecipitation and immunoblotting with p53 and plakoglobin antibodies. The results demonstrated that all p53 transfectants (WT, 175 and 273) co-precipitated plakoglobin. Similarly, plakoglobin co-precipitated the three forms of p53 in all transfectants, whereas control immunoprecipitates using mouse IgG were negative for both p53 and plakoglobin **(Fig 1B)**. Plakoglobin (PG) interactions with the three forms of p53 were also confirmed by GST pull-down assays. TCEs from untransfected H1299 cells (UT) and H1299 cells transiently expressing p53- (WT, 175 or 273) were mixed with PG-GST beads and processed for pull-down and immunoblotting with p53 antibodies. PG-GST beads pulled down p53 in all three transfectants **(Fig 1C, top)** whereas GST alone beads pulled down GST but not p53 **(Fig 1C, bottom)**, which further confirmed the interaction of p53 and plakoglobin. In addition, subcellular fractionation followed by immunoblotting with p53 and plakoglobin antibodies showed the presence of both proteins in the same subcellular compartments in all transfectants co-expressing p53 and plakoglobin (Fig 1D, * denotes non-specific band as discussed for Fig 1A).

**Fig 2:**
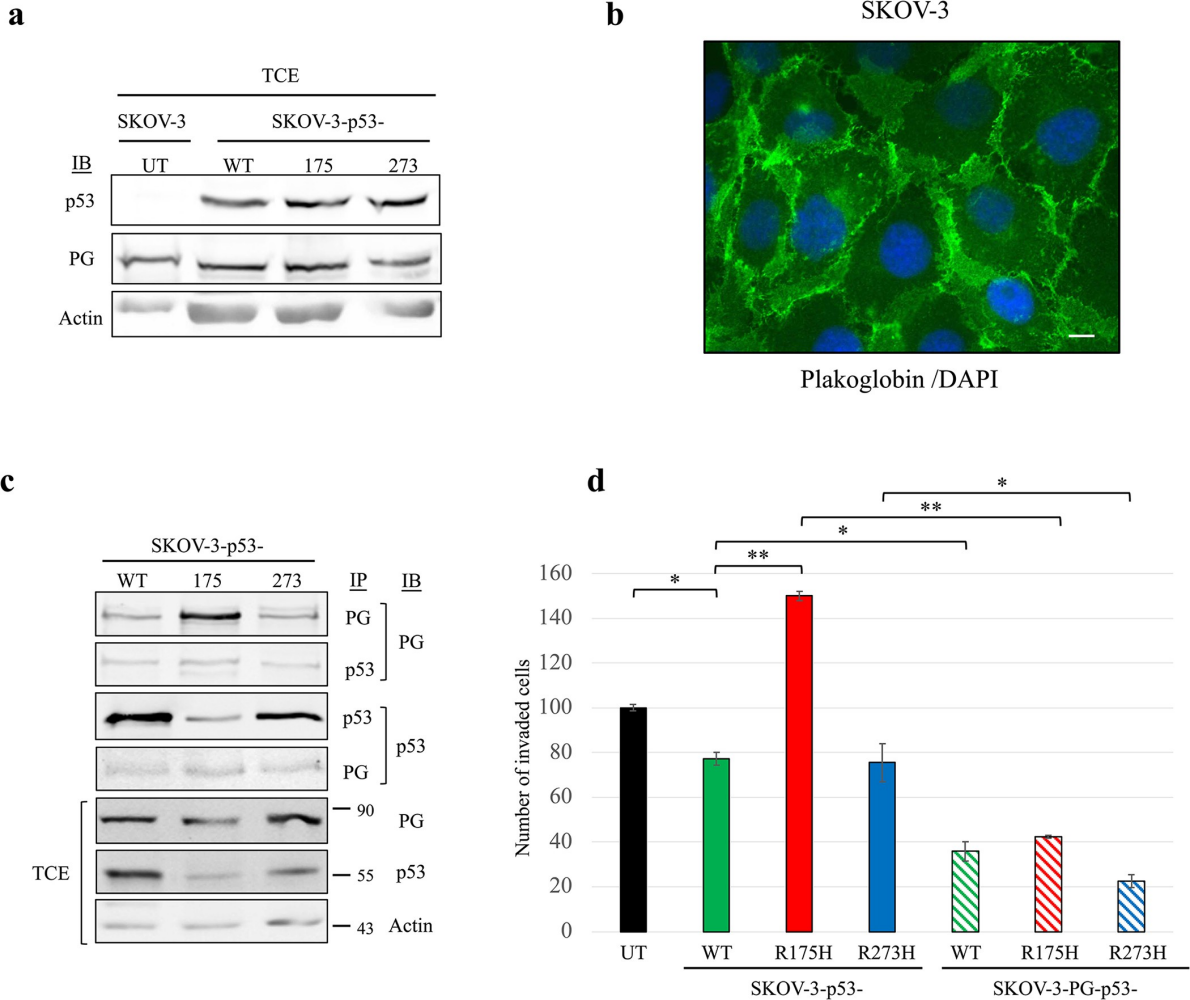
Plakoglobin (PG) interacted with p53- (WT, 175, 273) in SKOV-3 cells and reduced cellular invasion. **(a)** Total cellular extracts (TCEs) from untransfected (**UT**) SKOV-3 cells and SKOV-3 transfectants expressing p53- (WT, 175, 273) were processed for immunoblotting with p53 and plakoglobin antibodies. **(b)** SKOV-3 cells were grown on glass coverslip to confluency and processed for immunofluorescence staining using anti-plakoglobin antibody (green). Nuclei were counterstained with DAPI (blue). Bar, 40 μm. **(c)** TCEs from SKOV-3-p53- (WT, 175, 273) transfectants were processed for sequential reciprocal co-immunoprecipitation (**IP**) and immunoblotting (**IB**) using p53 and plakoglobin antibodies. Beta-actin was used as loading control. **(d)** Triplicate cultures of transfectants were processed for 24-hour transwell Matrigel invasion assays. Inserts were fixed, stained, and imaged under an inverted microscope at 20x magnification. The number of migrated or invaded cells was quantitated from five random fields using NIH ImageJ Cell Counter software. Results are presented as means ± SD in histograms. *p* values, * < 0.05, ** < 0.01.

To explore p53 and plakoglobin interaction in another cellular system under the same genetic background condition, we took advantage of SKOV-3 ovarian carcinoma cells that are p53 null but express endogenous plakoglobin **(Fig 2A)**. Immunofluorescence staining using plakoglobin antibody showed while it was primarily localized to the membrane it was also detected in the cytoplasm and nuclei **(Fig 2B)** We then examined for endogenous plakoglobin interaction with exogenously expressed p53- (WT, 175, 273). The results of sequential and reciprocal co-immunoprecipitation and immunoblotting of SKOV-3- p53- (WT, 175, 273) transfectants total cell extracts using p53 and plakoglobin antibodies showed endogenous plakoglobin interaction with all three p53 forms (p53- WT, 175, 273) **(Fig 2C)**. These results validate our previous observations and indicate that plakoglobin-p53 interaction is independent of tissue and cellular backgrounds that is, plakoglobin can still interact with all three forms of p53- (WT, 175, 273) irrespective of endogenous or exogenous expressive pattern or cellular background.

### Plakoglobin co-expression differentially affects the oncogenic properties (colony formation, migration and invasion) of p53 mutants

**Fig 3:**
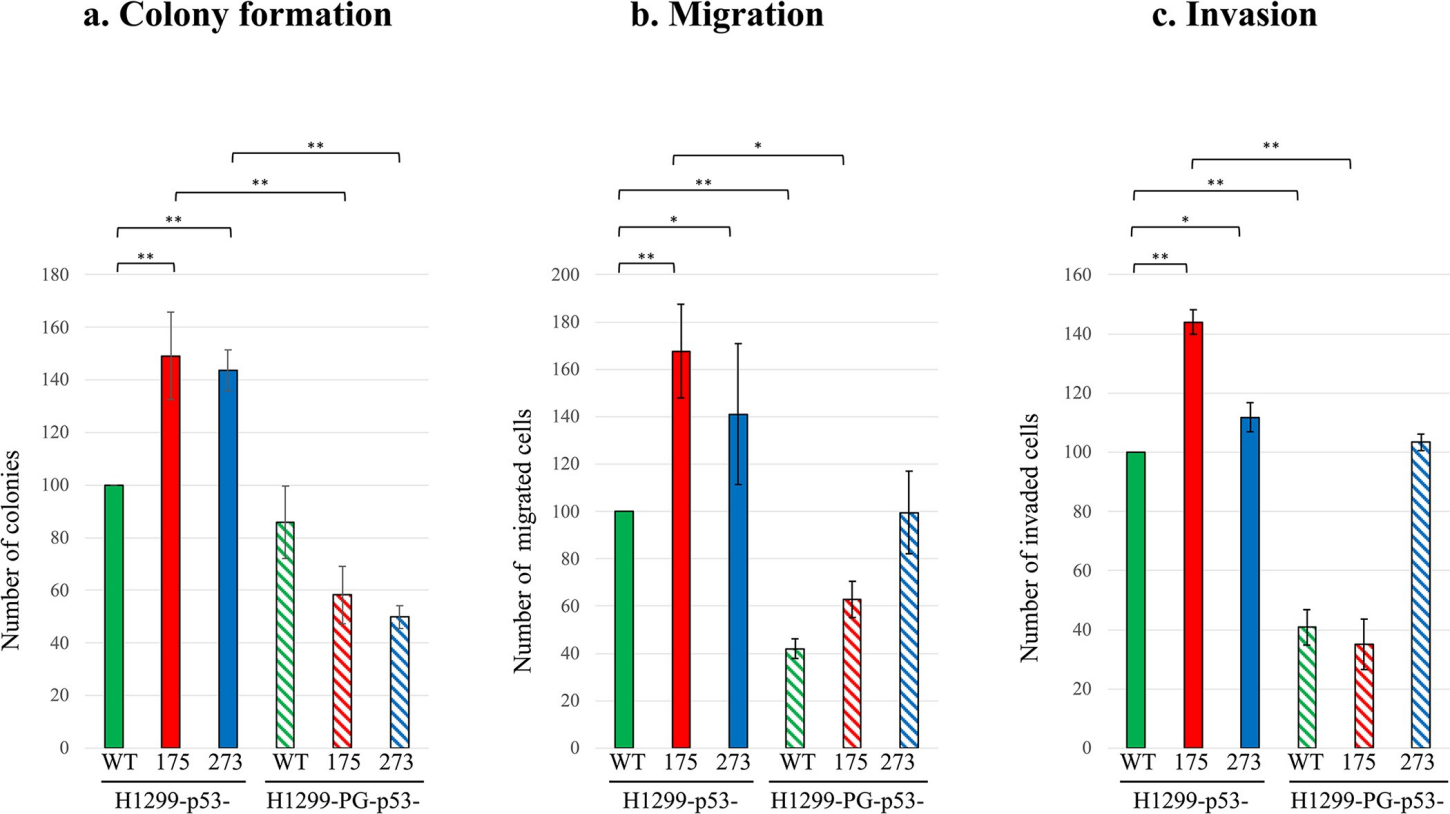
Colony formation, migration and invasion of H1299 cells expressing p53- (WT, R175H, R273H) without or with plakoglobin (PG). **(a)** H1299 cells transfectants were seeded in triplicates at 7.5x10^3^ cells in 35 mm dishes containing 0.35% top agar and 0.6% base agar and grew for two weeks. At the end of the two weeks, colonies were fixed, stained, counted. **(b)** and **(c)** Triplicate cultures of transfectants were processed for 24-hour transwell migration **(b)** and Matrigel invasion **(c)** assays. Inserts were fixed, stained, and imaged under an inverted microscope at 20x magnification. Results in all assays are presented in histograms as means ± SD after normalizing the values of the H1299 mutant p53 cells to p53-WT cells, and the values of the PG-p53 transfectants to their corresponding p53 only expressing cells. * p < 0.05, **p < 0.001.

To assess how plakoglobin expression affects the oncogenic properties of mutant p53- (175 and 273), we performed soft agar colony formation, migration, and invasion assays on H1299-p53- (WT, 175 and 273) transfectants independently or co-expressed with plakoglobin. When expressed individually in H1299 cells, p53-WT reduced colony formation by over 50%, whereas plakoglobin had no significant effect on colonogenicity **(S2 Fig)**. Compared with H1299-p53-WT cells, H1299-p53-175 and -273 cells exhibited increased colony formation by ~55% and 62%, respectively **(Fig 3A)**. Notably, co-expression of plakoglobin with p53-175 or -273 reduced colony formation by > 60% **(Fig 3A)**, indicating that plakoglobin can suppress colony formation induced by p53-175 and -273 in H1299 cells.

Likewise, when compared with p53-WT cells, both p53-175 and -273 transfectants showed increased cell migration (75% and 43%; **Fig 3B and S3A Fig**) and invasion (45% and 17%; **Fig 3C, S3B Fig**), respectively. Interestingly, co-expression of plakoglobin only decreased cell migration and invasion in p53-175 transfectants (35% and 65%, respectively) but had no significant effect on these properties in p53-273 cells (8% and 6%, respectively) **(Fig 3B and 3C, S3A and S3B Fig)**. These observations signified that plakoglobin expression exerted differential *in vitro* anti-tumorigenic effects in different p53 mutant (p53-175 and -273) backgrounds in H1299 cells; that is, plakoglobin reduced the colony formation properties of both H1299-p53-175 and -273 transfectants, but only reduced the invasive and migratory properties of H1299-p53-175 transfectants.

To confirm the anti-tumorigenic effects of plakoglobin on exogenously expressed p53 mutants, p53-175 and -273 mutants were transiently expressed in SKOV-3 ovarian carcinoma cells–which constitute a different cellular background to H1299 cells, and invasion assays were performed on the transfectants. We performed invasion assays only since *in vitro* cell invasion is a representative of the final stage of *in vivo* metastasis, following cell migration. Our results showed that only p53-175 expression significantly elevated cellular invasion relative to p53-WT in SKOV-3 cells. Exogenous expression of plakoglobin decreased cellular invasion in all transfectants [SKOV-3-p53- (WT, 175, 273)] **(Fig 2D).** Plakoglobin expression in H1299 cells [(H1299-PG-p53- (WT, 175, 273)] did not significantly decrease invasion in H1299-p53-273 transfectants **(Fig 3C)**, whereas exogenous expression of plakoglobin in SKOV-3 cells decreased cellular invasion in both SKOV-3-p53-175 and -273 cells **(Fig 2D)**.

In summary, these findings demonstrate that the functional effects of plakoglobin on mutant p53 tumorigenic abilities may be cell context dependent as seen from the tumor suppressive (inhibition of invasion and/or migration) functions of plakoglobin on p53-R175H but not p53-R273H in H1299 cells, and on both p53-R175H and p53-R273H in SKOV-3 cells.

### Plakoglobin co-expression alters *PUMA*, *BAX* and *S100A4* mRNA levels

To characterize the molecular players that may contribute to the observed differential anti-tumorigenic effects of plakoglobin on p53-R175H and -R273H, we investigated the effect of plakoglobin on the expression of two well-studied growth-regulating and proapoptotic p53-WT target genes–*PUMA* (p53 upregulated modulator of apoptosis) and *BAX* (Bcl2-associated X protein) [38–47] that is known to be dysregulated in mutant p53 cells. We also examined the effect of plakoglobin on the expression of S100A4, a tumor invasion and metastasis promoting protein known to enhance mutant p53 accumulation and oncogenic target gene expression in cancer cells [48, 49]. We selected these genes because mutant p53 have been shown to promote tumorigenesis by altering their expression [2, 6–9, 17, 18, 50] and earlier studies implicated an anti-tumorigenic role for plakoglobin via the regulation of their expression [24, 26, 27, 51–54].

**Fig 4:**
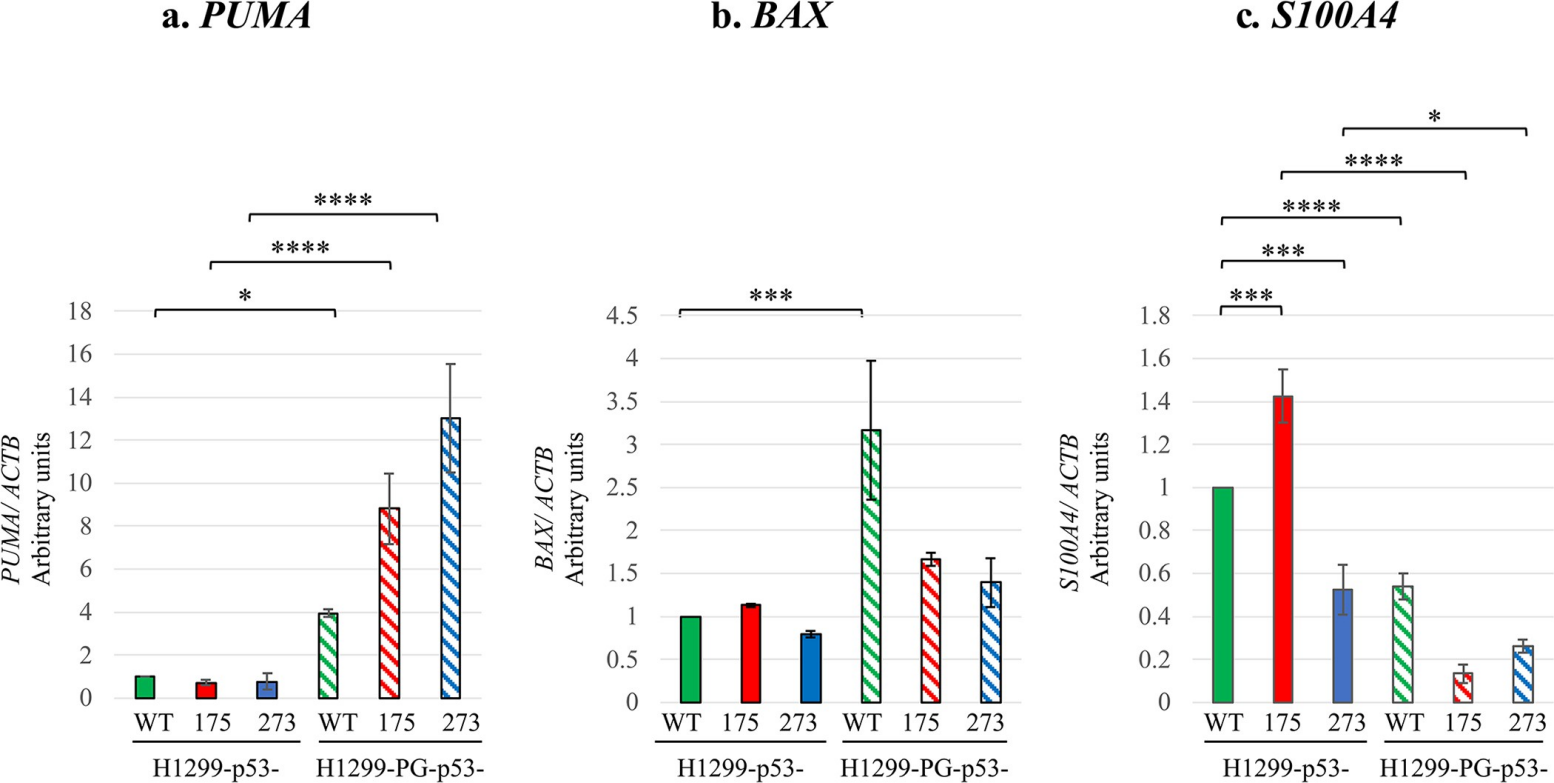
Changes in the mRNA expression of p53 and β-catenin target genes by plakoglobin co-expression. Total cellular RNA was extracted from confluent cultures of H1299 transfectants, reverse transcribed and processed for quantitative real-time PCR to detect ***PUMA* (a)**, ***BAX* (b)** and ***S100A4* (c) mRNAs**, using specific primers (**Table 2**). Target mRNA expression levels were normalized to the amount of ACTB mRNA in the same cell line. Then the resultant values for mutant p53- (175, 273) transfectants were normalized to p53-WT cells, whereas the values for PG-p53 transfectants to their p53 only expressing counterparts. Histograms were then constructed based on the mean ± SD values. *p < 0.05, **p < 0.01, ***p < 0.001, ****p < 0.0001.

We first assessed the effects of individual expression of p53-WT or plakoglobin on *PUMA*, *BAX* and *S100A4* expression in H1299 cells. We found that while p53-WT did not affect the expression of *PUMA*, *BAX* and *S100A4*, plakoglobin significantly increased *PUMA* and *BAX* but not *S100A4* expression in H1299 cells **(S4A–S4C Fig)**. These studies were done in the absence of cellular stresses such as DNA damage or staurosporine treatment, which explains why *PUMA* and *BAX* expression was not induced in response to p53-WT expression in H1299 cells [29, 55, 56]. Expression of mutant p53-(175, 273) in H1299 cells also did not affect the expression of *PUMA* or *BAX* transcripts. When co-expressed, plakoglobin resulted in a significant increase in *PUMA* but not *BAX* expression in H1299-p53-175 and -273 cells **(Fig 4A and 4B)** and notably, a significant increase in *PUMA* and *BAX* expression in control H1299-p53-WT cells **(Fig 4A and 4B)**. This indicates a role for plakoglobin in the modulation of *PUMA* and *BAX* expression in p53-WT as well as mutant (p53-175 and -273) expressing cells even in the absence of cellular stresses such as DNA damage [29, 55, 56].

For *S100A4*, we found that p53-175 significantly increased while p53-273 significantly decreased *S100A4* transcripts level in H1299 cells relative to p53-WT. Interestingly, the co-expression of plakoglobin resulted in a significant decrease in *S100A4* levels in both H1299-p53-175 and -273 transfectants **(Fig 4C)**, and control H1299-p53-WT cells **(Fig 4C)**. Our findings again suggest a role for plakoglobin in the modulation of *S100A4* expression in both p53-WT and mutant (p53-175 and -273)- expressing cells. Together, our findings demonstrate that plakoglobin has a mutant p53-independent effect as seen by the significantly increased *PUMA* and *BAX* transcript levels in both H1299-p53-WT and mutant (p53-175, 273) cells. Conversely, plakoglobin has a p53-dependent effect (whether wild-type or mutant) on *S100A4* expression as evidenced by the decreased trend of *S100A4* expression when plakoglobin was co-expressed in either H1299- p53- (WT, 175, or 273) cells.

### Plakoglobin decreases nuclear β-catenin and increases nuclear nucleophosmin (NPM) levels in mutant p53-expressing H1299 cells

**Fig 5:**
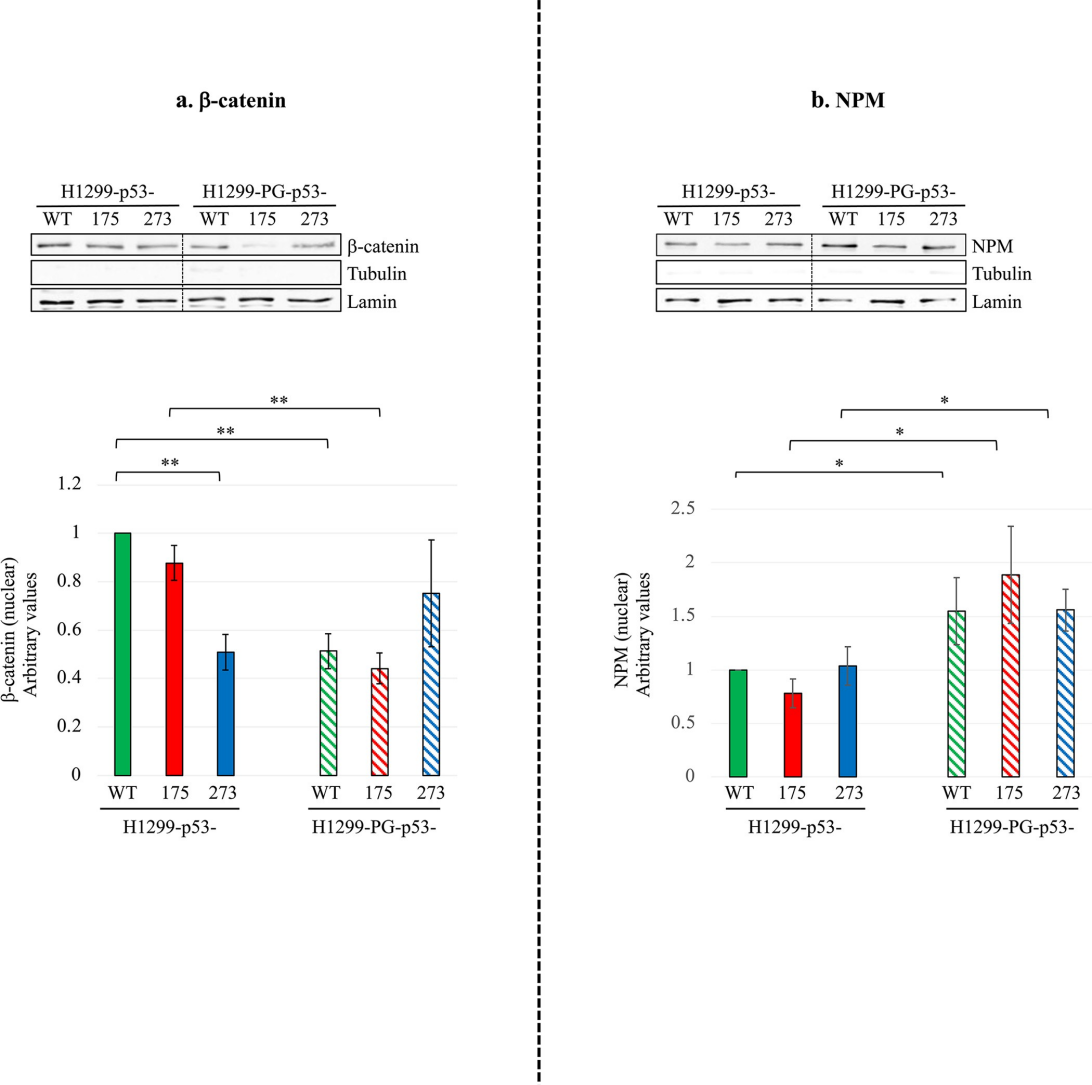
Plakoglobin differentially affects the nuclear level of β-catenin and nucleophosmin (NPM) in H1299 cells expressing p53- (WT, R175H, R273H) without or with plakoglobin (PG). Equal amounts of nuclear extracts from various transfectants were processed for immunoblotting using **β-catenin (a) and NPM (b).** H1299- p53 and H1299-PG-p53 were run on the same gel separated by a lane. The purity of the nuclear fraction was confirmed by immunoblotting with tubulin and lamin antibodies, respectively, which also served as loading controls. Immunoblots representative of the nuclear distribution of β-catenin **(a)** and NPM **(b)** in various transfectants. Lamin and tubulin blots for NPM were run on different percentage gels using the same lysates from the same experiment. All blots were quantitated by NIH Image software and histograms constructed, which represent means ± SD of at least 3 independent experiments after normalizing the values of the H1299 mutant p53 cells to p53-WT cells, and the values of the PG-p53 transfectants to their corresponding p53 only expressing cells. *p < 0.05, **p < 0.01.

We next investigated the effects of plakoglobin on the subcellular distribution of β-catenin and nucleophosmin (NPM) that have been linked to plakoglobin anti-tumor roles in cancer cells [35, 57–59]. Indeed, work from our laboratory and others have previously demonstrated that a potential mechanism of plakoglobin tumor-suppressive function is the regulation of β-catenin subcellular distribution and oncogenic activities [35, 37, 52, 57, 58, 60, 61]. We have also shown that plakoglobin expression in the mutant p53 (R280K)- expressing and highly invasive breast carcinoma cells MDA-MB-231 increased nuclear NPM level and this redistribution was concurrent with the decreased *in vitro* tumorigenic properties of these cells **[**[58] **Figs 4 and 5 therein]**.

Analysis of the effect of the p53 mutants on the subcellular distribution of β-catenin and NPM showed that while p53-175 had no significant effect on the level of nuclear β-catenin in H1299 cells, p53-273 significantly decreased (~50%) nuclear β-catenin expression in H1299 cells relative to H1299-p53-WT cells. When co-expressed, plakoglobin resulted in a significant reduction (60%) in nuclear β-catenin levels in H1299-p53-175 and control p53-WT cells. Contrastingly, though not statistically significant, we observed an increase in nuclear β-catenin levels upon co-expression of plakoglobin in H1299-p53-273 transfectants **(Fig 5A)**. We interpret these results as confirming a role for plakoglobin in reducing the nuclear accumulation of β-catenin in p53-175 but not p53-273 transfectants. It appears plakoglobin’s effect on nuclear β-catenin levels is dependent on its association with p53-WT and p53-175 but not p53-273.

With regard to NPM, we found that the levels of nuclear NPM were similar in all H1299-p53- (WT, 175, 273) transfectants. When co-expressed, plakoglobin increased the nuclear pool of NPM by 89% and 55% in H1299-p53-175 and -273 cells, respectively **(Fig 5B)**. We also observed a significant increase (54%) in the nuclear pool of NPM in H1299-p53-WT cells when plakoglobin was co-expressed in these cells **(Fig 5B)**. It is important to note that we also found plakoglobin in complex with NPM in all H1299-PG-p53- (WT, 175, 273) transfectants (**S5 Fig),** indicating that plakoglobin interaction with the three forms of p53 increases the nuclear NPM; and that neither p53-175 nor p53-273 mutations can alter this activity. Since increased nuclear β-catenin and decreased nuclear NPM have generally been associated with tumorigenic properties [26, 58], our findings provide a potential mechanism via which plakoglobin may exert its tumor suppressor function in these cells.

## Discussion

We have previously shown that plakoglobin interacts with endogenously expressed p53-WT and p53 mutants and that exogenous expression of plakoglobin in plakoglobin deficient and mutant p53 (e.g., R280K, R273H, S241F, S215R) expressing carcinoma cell lines of different origins restored p53 tumor suppressor activity *in vitro* [24–28]. Herein, we show that exogenously expressed plakoglobin interacted with both exogenously expressed p53^R175H^ conformational and p53^R273H^ contact mutants in the plakoglobin-deficient and p53-null H1299 cells and differentially affected their oncogenic functions *in vitro*. Specifically, plakoglobin co-expression significantly reduced the tumorigenic (colony formation, migratory and invasiveness) properties of H1299-p53-175 cells, whereas it minimally affected the tumorigenic (migratory and invasiveness) properties of H1299-p53-273 cells. It is important to mention that we observed a more tumorigenic effect of p53-175 compared to p53-273 in H1299 cells. That is, H1299-p53-175 cells exhibited significantly increased migratory and invasive properties relative to their p53-273 expressing counterpart. We also showed that endogenously expressed plakoglobin in the p53 null SKOV-3 mammary carcinoma cells interacted with exogenously expressed p53-175 and p53-273 mutants in these cells and resulted in a similar reduction of the invasive properties of both p53-mutants in SKOV-3 cells. These observations indicate that the tumor suppressive effects of plakoglobin on oncogenic functions of p53-175 and -273 mutants are cell context dependent. Further supporting the necessity of performing functional comparative analysis of different mutants in the same genetic background.

Several studies have demonstrated that the oncogenic properties of p53 mutants are mediated in part by their altered transcriptional effects on p53-WT target genes. Accordingly, we found that both p53-175 and p53-273 had no effect on the transcription of the p53-WT target genes *PUMA* and *BAX*, which is consistent with findings in other studies [29, 55, 56]. Interestingly, plakoglobin co-expression in H1299-p53-175 and -273 cells significantly increased *PUMA* but not *BAX* transcript levels in both the p53-175 and p53-273 transfectants suggesting a direct regulation of *PUMA* but not *BAX* transcripts by plakoglobin in H1299-p53-175 and -273 cells. *PUMA* is a well-known proapoptotic/anti-tumorigenic p53-WT target gene [41, 42, 46]. Its increase in both H1299-p53-175 and p53-273 cells by plakoglobin may explain how plakoglobin was able to inhibit the colony formation features displayed by both transfectants in H1299 cells. It’s noteworthy to mention that plakoglobin expression alone did induced elevated *PUMA* level, and this effect was further increased in the p53-175 or p53-273 transfectants **(S4 Fig)**. While although, plakoglobin expression alone also resulted in elevated *BAX* level, this effect was dampened by p53-175 or p53-273 respective co-expression **(S4 Fig)**. These observations suggest that plakoglobin’s effects on *PUMA* and *BAX* expression is mutant p53-specific since WT-p53 does not interfere with plakoglobin’s effect on *PUMA* and *BAX* levels.

Investigation of a different gene, *S100A4* that is known to promote mutant p53 accumulation and oncogenic target gene expression in cancer cells, revealed that *S100A4* is significantly increased by p53-175 but not p53-273 in H1299 cells. S100A4 is a well-known promoter of cancer cell migration, invasion and metastasis [62, 63]. As such, its upregulated expression in response to p53-175 but not p53-273 in H1299 cells may be responsible for the increased migratory and invasive properties exhibited by the H1299-p53-175 relative to -273 in these cells. Importantly, the co-expression of plakoglobin appreciably reduced *S100A4* transcript levels in the p53-175 cells, which may explain plakoglobin’s suppressive effect on the migration and invasion of H1299-p53-175 cells.

A further interesting finding in this study is that plakoglobin co-expression differentially affected the levels of nuclear β-catenin, which is pro-tumorigenic in the H1299-p53-175 and p53-273 transfectants. While a significant decline in nuclear β-catenin levels was observed in the H1299-PG-p53-175 cells there was a slight increase in nuclear β-catenin levels in the H1299-PG-p53-273 cells, though it was not significant. Plakoglobin suppression of nuclear β-catenin localization in the H1299-PG-p53-175 cells could explain its anti-tumorigenic effect in these cells. Its lack of inhibition of the nuclear β-catenin levels in the H1299-PG-p53-273 cells could also explain why plakoglobin had no significant effect on the migratory and invasive properties displayed by the H1299-PG-p53-273 cells. Another notable finding was the increased nuclear pool of NPM upon plakoglobin co-expression in H1299-p53-175- and -273 cells. Nuclear NPM localization/levels have been associated with decreased tumorigenic properties *in vitro*–specifically decreased growth of mutant p53-expressing cells [58]. This may thus further explain the suppressive effects of plakoglobin on the colony formation/anchorage-independent growth of H1299-p53-175- and -273 cells.

**Fig 6:**
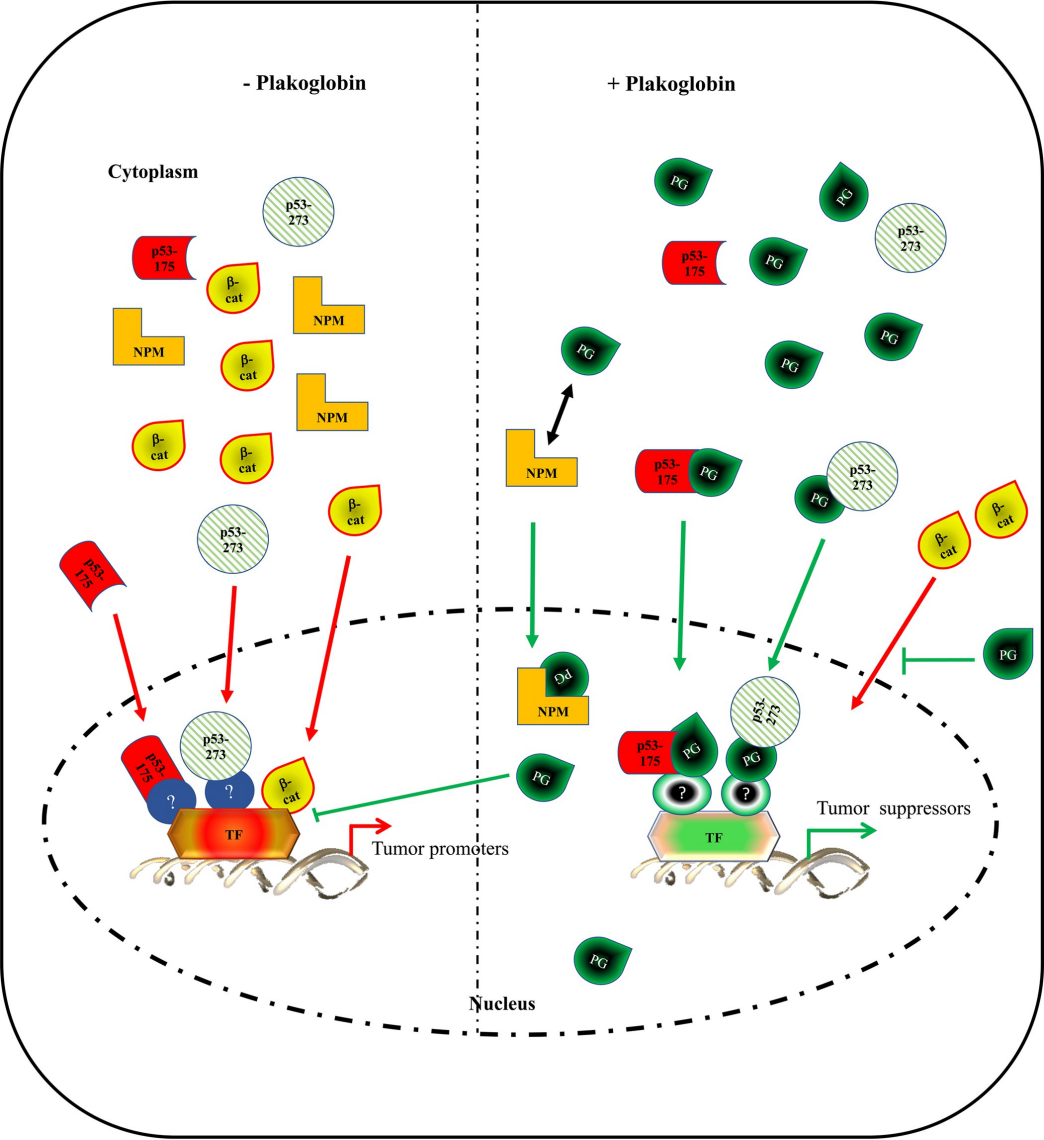
A potential model of plakoglobin tumor suppressor activity via regulation of gene expression and cell signaling in a p53-independent and p53-dependent manner. In the absence of plakoglobin, mutant p53-R175H or p53-R273H can translocate to the nucleus and interact with transcription factors to activate expression of gene products associated with tumorigenesis. Under this condition, there is a reduced level of nuclear nucleophosmin and an increased level of nuclear β-catenin that promote tumorigenesis. When expressed, plakoglobin shuttles in and out of the nucleus. In the cytoplasm, plakoglobin reduces β-catenin level and its translocation into the nucleus/oncogenic activation. Cytoplasmic plakoglobin can also interact with NPM and p53-R175H and p53-R273H and the resulting plakoglobin containing complexes translocate into the nucleus. This increases nuclear NPM that promotes its tumor suppressive activity. In addition, nuclear PG-mutant p53 complexes may interact with different set of transcription factors that activate the expression of genes involved in tumor suppression. Furthermore, nuclear plakoglobin also inhibits β-catenin/TCF and activation of the tumor promoting Wnt target genes.

Together with our previous studies, our results indicate that plakoglobin represses the oncogenic functions of the p53-175 and p53-273 mutants investigated in this study differentially and by at least two mechanisms: **(1)** Modulation of gene transcription including targets such as *PUMA* and *S100A4* and **(2)** Regulation of the nuclear localization/levels of β-catenin and NPM. **Fig 6** is a conceptual model of how plakoglobin may act as a tumor suppressor by interacting with and regulating p53 (mutants) tumorigenic functions. In this model, we postulate that in the absence of plakoglobin, mutant p53 (175 and 273) and β-catenin translocate into the nucleus and interact with oncogenic transcription factors to activate gene expression associated with tumorigenesis. The presence of plakoglobin decreases tumor-promoting transcriptional events, by several potential and non-mutually exclusive mechanisms: 1) Plakoglobin binds to mutant p53 directly and the complex translocates into the nucleus where it interacts with tumor inhibitory transcription factors. Recent studies have demonstrated that small molecules /drugs that can interact with p53 mutants to restore their tumor suppressor activities are more effective than deleting/inactivating the mutants [64, 65]. 2) Plakoglobin modulates oncogenic activities of β-catenin by either promoting its degradation in the cytoplasm or inhibiting its interaction with tumor promoting transcription factors. 3) Plakoglobin interacts with NPM and increases NPM nuclear localization and tumor suppression phenotype. Additionally, plakoglobin may directly regulate the activation of certain genes, such as *PUMA* and *BAX* (**S4 Fig**). While plakoglobin functions are not affected by p53-WT, it is modulated by p53-175 and p53-273 in a target-specific manner (**Fig 4**), that is both *PUMA* and *BAX* level are in increased in H1299-PG cells (**S4A and S4B Fig),** whereas *PUMA* level is further increased, while *BAX* level is decreased in H1299- [PG-p53-175 and PG-p53-273] transfectants (**Fig 4A and 4B**).

## Conclusion

Overall, our results signify that plakoglobin can reactivate the tumor suppressive functions of p53-R273H and p53-R175H mutants in cells of the same genetic background, albeit to a different extent. Plakoglobin can thus potentially be used for the development of effective therapeutic strategies in highly aggressive mutant p53-expressing cancer cells. Using *in silico* modelling, we have determined the 3-D structure of PG-p53-WT and PG-p53^R175H^, identified the amino acid residues involved in their interaction and validated the role of these residues in the plakoglobin- p53-R175H interaction [65]. Information derived from these studies can be utilized to develop compounds that mimic plakoglobin-p53 interaction, restore tumor suppressor function of mutants and have the potential to function as therapeutics in various cancers [64]. These compounds will further have the advantage of representing a naturally occurring biological interaction that positively promotes p53 tumor/metastasis suppressor function, potentially, with fewer side effects in patients with mutant p53-expressing tumors.

## Supporting information

S1 Raw images (PDF)

S1 Raw data (XLSX)

S1 Fig Plakoglobin (PG) expression and localization in H1299 parental cells and H1299-PG transfectants. H1299 and H1299 transfectants were processed for immunofluorescence and immunoblotting using plakoglobin (PG), p53 and acting (loading control) antibodies. UT, untransfected; VT (vector transfected).
(TIF)

S2 Fig Colony formation of H1299, H1299-p53-WT and H1299-plakoglobin (PG) cells. H1299 cells and H1299 transfectants were seeded in triplicates at 7.5x10^3^ cells in 35 mm dishes containing 0.35% top agar and 0.6% base agar and grew for two weeks. At the end of the two weeks, colonies were fixed, stained, counted and the results presented as means ± SD. *p*, ** < 0.01.
(TIF)

S3 Fig Migration and invasion of 111299 cells expressing p53- (WT, R175H, R273H) without (-PG) or with (+PG) plakoglobin. Triplicate cultures of transfectants were processed for 24-hour transwell migration **(a)** and Matrigel invasion **(b)** assays. Inserts were fixed, stained, and imaged under an inverted microscope at 20x magnification.
(TIF)

S4 Fig Changes in the mRNA levels of p53 or f3-catenin target genes in H1299 cells expressing p53 or plakoglobin (PG). Total cellular RNA was extracted from confluent cultures of H1299, H1299-p53 and H1299-PG cells, reverse transcribed and processed for quantitative real-time PCR to detect ***PUMA* (a)**, ***BAX* (b)** and ***S100A4* (c) mRNAs**, using specific primers (**Table 2**). Expression levels were normalized to the amount of *ACTS mRNA* in the same cell line. Histograms were constructed based on the mean ± SD. The values of transfectants were normalized to that of the untransfected H1299 cells. **p < 0.001, ****p < 0.0001.
(TIF)

S5 Fig Nucleophosmin (NPM) interacted with plakoglobin (PG) in H1299-p53-(WT, 175, 273) transfectants. Total cellular extracts (TCEs) from H1299-PG-p53-(WT, 175, 273) cells were processed for sequential co-immunoprecipitation (**IP**) and immunoblotting (**IB**) with nucleophosmin, plakoglobin and p53 antibodies. Beta-actin was used as loading control.
(TIF)

## List of abbreviations

PG: plakoglobi
WT: wild-type
GST: glutathione S-transferase
NPM: nucleophosm
PUMA: p53 upregulated modulator of apoptosis
BAX: Bcl2-associated X protein

## References

[1] Lane DP. Cancer. p53, guardian of the genome. Nature. 1992;358(6381):15–6. doi: 10.1038/358015a0 1614522

[2] Abdel-Magid AF. Reactivation of the Guardian of the Genome P53: A Promising Strategy for Treatment of Cancer. ACS Med Chem Lett. 2021;12(3):331–3. doi: 10.1021/acsmedchemlett.1c00098 33738057 PMC7957941

[3] Yamamoto S, Iwakuma T. Regulators of Oncogenic Mutant TP53 Gain of Function. Cancers (Basel). 2018;11(1). doi: 10.3390/cancers11010004 30577483 PMC6356290

[4] Sabapathy K, Lane DP. Therapeutic targeting of p53: all mutants are equal, but some mutants are more equal than others. Nat Rev Clin Oncol. 2018;15(1):13–30. doi: 10.1038/nrclinonc.2017.151 28948977

[5] Peuget S, Zhou X, Selivanova G. Translating p53-based therapies for cancer into the clinic. Nat Rev Cancer. 2024. doi: 10.1038/s41568-023-00658-3 38287107

[6] Wang H, Liao P, Zeng SX, Lu H. It takes a team: a gain-of-function story of p53-R249S. J Mol Cell Biol. 2019;11(4):277–83. doi: 10.1093/jmcb/mjy086 30608603 PMC6487778

[7] Alvarado-Ortiz E, de la Cruz-López KG, Becerril-Rico J, Sarabia-Sánchez MA, Ortiz-Sánchez E, García-Carrancá A. Mutant p53 Gain-of-Function: Role in Cancer Development, Progression, and Therapeutic Approaches. Front Cell Dev Biol. 2020;8:607670. doi: 10.3389/fcell.2020.607670 33644030 PMC7905058

[8] Stein Y, Aloni-Grinstein R, Rotter V. Mutant p53 oncogenicity: dominant-negative or gain-of-function? Carcinogenesis. 2020;41(12):1635–47. doi: 10.1093/carcin/bgaa117 33159515

[9] Stein Y, Rotter V, Aloni-Grinstein R. Gain-of-Function Mutant p53: All the Roads Lead to Tumorigenesis. Int J Mol Sci. 2019;20(24). doi: 10.3390/ijms20246197 31817996 PMC6940767

[10] Moxley AH, Reisman D. Context is key: Understanding the regulation, functional control, and activities of the p53 tumour suppressor. Cell Biochem Funct. 2021;39(2):235–47. doi: 10.1002/cbf.3590 32996618

[11] Tang Q, Su Z, Gu W, Rustgi AK. Mutant p53 on the Path to Metastasis. Trends Cancer. 2020;6(1):62–73. 10.1016/j.trecan.2019.11.004 PMC7485681 31952783

[12] Pitolli C, Wang Y, Mancini M, Shi Y, Melino G, Amelio I. Do Mutations Turn p53 into an Oncogene? Int J Mol Sci. 2019;20(24). doi: 10.3390/ijms20246241 31835684 PMC6940991

[13] Midgley CA, Lane DP. p53 protein stability in tumour cells is not determined by mutation but is dependent on Mdm2 binding. Oncogene. 1997;15(10):1179–89. doi: 10.1038/sj.onc.1201459 9294611

[14] Terzian T, Suh YA, Iwakuma T, Post SM, Neumann M, Lang GA, et al. The inherent instability of mutant p53 is alleviated by Mdm2 or p16INK4a loss. Genes Dev. 2008;22(10):1337–44. doi: 10.1101/gad.1662908 18483220 PMC2377188

[15] Frum RA, Grossman SR. Mechanisms of mutant p53 stabilization in cancer. Subcell Biochem. 2014;85:187–97. doi: 10.1007/978-94-017-9211-0_10 25201195

[16] Billant O, Friocourt G, Roux P, Voisset C. p53, A Victim of the Prion Fashion. Cancers (Basel). 2021;13(2). doi: 10.3390/cancers13020269 33450819 PMC7828285

[17] Baugh EH, Ke H, Levine AJ, Bonneau RA, Chan CS. Why are there hotspot mutations in the TP53 gene in human cancers? Cell Death Differ. 2018;25(1):154–60. doi: 10.1038/cdd.2017.180 29099487 PMC5729536

[18] Bouaoun L, Sonkin D, Ardin M, Hollstein M, Byrnes G, Zavadil J, et al. TP53 Variations in Human Cancers: New Lessons from the IARC TP53 Database and Genomics Data. Hum Mutat. 2016;37(9):865–76. doi: 10.1002/humu.23035 27328919

[19] Zhou X, Hao Q, Lu H. Mutant p53 in cancer therapy-the barrier or the path. J Mol Cell Biol. 2019;11(4):293–305. doi: 10.1093/jmcb/mjy072 30508182 PMC6487791

[20] Kastenhuber ER, Lowe SW. Putting p53 in Context. Cell. 2017;170(6):1062–78. doi: 10.1016/j.cell.2017.08.028 28886379 PMC5743327

[21] Brady CA, Attardi LD. p53 at a glance. J Cell Sci. 2010;123(Pt 15):2527–32. doi: 10.1242/jcs.064501 20940128 PMC2912460

[22] Mello SS, Attardi LD. Not all p53 gain-of-function mutants are created equal. Cell Death Differ. 2013;20(7):855–7. doi: 10.1038/cdd.2013.53 23749181 PMC3679467

[23] Brady SW, Gout AM, Zhang J. Therapeutic and prognostic insights from the analysis of cancer mutational signatures. Trends Genet. 2022;38(2):194–208. doi: 10.1016/j.tig.2021.08.007 34483003 PMC8752466

[24] Aktary Z, Kulak S, Mackey J, Jahroudi N, Pasdar M. Plakoglobin interacts with the transcription factor p53 and regulates the expression of 14-3-3σ. J Cell Sci. 2013;126(Pt 14):3031–42. 23687381 10.1242/jcs.120642

[25] Alaee M, Padda A, Mehrabani V, Churchill L, Pasdar M. The physical interaction of p53 and plakoglobin is necessary for their synergistic inhibition of migration and invasion. Oncotarget. 2016;7(18):26898–915. doi: 10.18632/oncotarget.8616 27058623 PMC5042024

[26] Alaee M, Nool K, Pasdar M. Plakoglobin restores tumor suppressor activity of p53. Cancer Sci. 2018;109(6):1876–88. 29660231 10.1111/cas.13612 PMC5989865

[27] Aktary Z, Pasdar M. Plakoglobin represses SATB1 expression and decreases in vitro proliferation, migration and invasion. PLoS One. 2013;8(11):e78388. doi: 10.1371/journal.pone.0078388 24260116 PMC3832639

[28] Alaee M, Danesh G, Pasdar M. Plakoglobin Reduces the in vitro Growth, Migration and Invasion of Ovarian Cancer Cells Expressing N-Cadherin and Mutant p53. PLoS One. 2016;11(5):e0154323. doi: 10.1371/journal.pone.0154323 27144941 PMC4856367

[29] Tan BS, Tiong KH, Choo HL, Chung FF, Hii LW, Tan SH, et al. Mutant p53-R273H mediates cancer cell survival and anoikis resistance through AKT-dependent suppression of BCL2-modifying factor (BMF). Cell Death Dis. 2015;6:e1826. doi: 10.1038/cddis.2015.191 26181206 PMC4650736

[30] Muller PA, Caswell PT, Doyle B, Iwanicki MP, Tan EH, Karim S, et al. Mutant p53 drives invasion by promoting integrin recycling. Cell. 2009;139(7):1327–41. doi: 10.1016/j.cell.2009.11.026 20064378

[31] Mukherjee S, Maddalena M, Lü Y, Martinez S, Nataraj NB, Noronha A, et al. Cross-talk between mutant p53 and p62/SQSTM1 augments cancer cell migration by promoting the degradation of cell adhesion proteins. Proc Natl Acad Sci U S A. 2022;119(17):e2119644119. doi: 10.1073/pnas.2119644119 35439056 PMC9173583

[32] Hassin O, Nataraj NB, Shreberk-Shaked M, Aylon Y, Yaeger R, Fontemaggi G, et al. Different hotspot p53 mutants exert distinct phenotypes and predict outcome of colorectal cancer patients. Nat Commun. 2022;13(1):2800. doi: 10.1038/s41467-022-30481-7 35589715 PMC9120190

[33] Lv T, Wu X, Sun L, Hu Q, Wan Y, Wang L, et al. p53-R273H upregulates neuropilin-2 to promote cell mobility and tumor metastasis. Cell Death Dis. 2017;8(8):e2995. doi: 10.1038/cddis.2017.376 28796261 PMC5596564

[34] Kadosh E, Snir-Alkalay I, Venkatachalam A, May S, Lasry A, Elyada E, et al. The gut microbiome switches mutant p53 from tumour-suppressive to oncogenic. Nature. 2020;586(7827):133–8. doi: 10.1038/s41586-020-2541-0 32728212 PMC7116712

[35] Li L, Chapman K, Hu X, Wong A, Pasdar M. Modulation of the oncogenic potential of beta-catenin by the subcellular distribution of plakoglobin. Mol Carcinog. 2007;46(10):824–38. doi: 10.1002/mc.20310 17415780

[36] Valenti F, Ganci F, Fontemaggi G, Sacconi A, Strano S, Blandino G, et al. Gain of function mutant p53 proteins cooperate with E2F4 to transcriptionally downregulate RAD17 and BRCA1 gene expression. Oncotarget. 2015;6(8):5547–66. doi: 10.18632/oncotarget.2587 25650659 PMC4467386

[37] Choi HJ, Gross JC, Pokutta S, Weis WI. Interactions of plakoglobin and beta-catenin with desmosomal cadherins: basis of selective exclusion of alpha- and beta-catenin from desmosomes. J Biol Chem. 2009;284(46):31776–88. doi: 10.1074/jbc.M109.047928 19759396 PMC2797248

[38] Lees A, Sessler T, McDade S. Dying to Survive-The p53 Paradox. Cancers (Basel). 2021;13(13). 10.3390/cancers13133257 PMC8268032 34209840

[39] Bouvard V, Zaitchouk T, Vacher M, Duthu A, Canivet M, Choisy-Rossi C, et al. Tissue and cell-specific expression of the p53-target genes: bax, fas, mdm2 and waf1/p21, before and following ionising irradiation in mice. Oncogene. 2000;19(5):649–60. doi: 10.1038/sj.onc.1203366 10698510

[40] Miyashita T, Reed JC. Tumor suppressor p53 is a direct transcriptional activator of the human bax gene. Cell. 1995;80(2):293–9. doi: 10.1016/0092-8674(95)90412-3 7834749

[41] Nakano K, Vousden KH. PUMA, a novel proapoptotic gene, is induced by p53. Mol Cell. 2001;7(3):683–94. doi: 10.1016/s1097-2765(01)00214-3 11463392

[42] Hikisz P, Kiliańska ZM. PUMA, a critical mediator of cell death—one decade on from its discovery. Cell Mol Biol Lett. 2012;17(4):646–69. doi: 10.2478/s11658-012-0032-5 23001513 PMC6275950

[43] Beyfuss K, Hood DA. A systematic review of p53 regulation of oxidative stress in skeletal muscle. Redox Rep. 2018;23(1):100–17. doi: 10.1080/13510002.2017.1416773 29298131 PMC6748683

[44] Hernández Borrero LJ, El-Deiry WS. Tumor suppressor p53: Biology, signaling pathways, and therapeutic targeting. Biochim Biophys Acta Rev Cancer. 2021;1876(1):188556. doi: 10.1016/j.bbcan.2021.188556 33932560 PMC8730328

[45] Wu X, Deng Y. Bax and BH3-domain-only proteins in p53-mediated apoptosis. Front Biosci. 2002;7:d151–6. doi: 10.2741/A772 11779719

[46] Sinha S, Malonia SK, Mittal SP, Singh K, Kadreppa S, Kamat R, et al. Coordinated regulation of p53 apoptotic targets BAX and PUMA by SMAR1 through an identical MAR element. EMBO J. 2010;29(4):830–42. doi: 10.1038/emboj.2009.395 20075864 PMC2829167

[47] Willis SN, Adams JM. Life in the balance: how BH3-only proteins induce apoptosis. Curr Opin Cell Biol. 2005;17(6):617–25. doi: 10.1016/j.ceb.2005.10.001 16243507 PMC2930980

[48] Bjørnland K, Winberg JO, Odegaard OT, Hovig E, Loennechen T, Aasen AO, et al. S100A4 involvement in metastasis: deregulation of matrix metalloproteinases and tissue inhibitors of matrix metalloproteinases in osteosarcoma cells transfected with an anti-S100A4 ribozyme. Cancer Res. 1999;59(18):4702–8. 10493528

[49] Shen W, Chen D, Liu S, Chen L, Yu A, Fu H, et al. S100A4 interacts with mutant p53 and affects gastric cancer MKN1 cell autophagy and differentiation. Int J Oncol. 2015;47(6):2123–30. doi: 10.3892/ijo.2015.3209 26497012

[50] Goh AM, Coffill CR, Lane DP. The role of mutant p53 in human cancer. J Pathol. 2011;223(2):116–26. doi: 10.1002/path.2784 21125670

[51] Charpentier E, Lavker RM, Acquista E, Cowin P. Plakoglobin suppresses epithelial proliferation and hair growth in vivo. J Cell Biol. 2000;149(2):503–20. doi: 10.1083/jcb.149.2.503 10769039 PMC2175163

[52] Hakimelahi S, Parker HR, Gilchrist AJ, Barry M, Li Z, Bleackley RC, et al. Plakoglobin regulates the expression of the anti-apoptotic protein BCL-2. J Biol Chem. 2000;275(15):10905–11. doi: 10.1074/jbc.275.15.10905 10753888

[53] Dusek RL, Godsel LM, Chen F, Strohecker AM, Getsios S, Harmon R, et al. Plakoglobin deficiency protects keratinocytes from apoptosis. J Invest Dermatol. 2007;127(4):792–801. doi: 10.1038/sj.jid.5700615 17110936

[54] Xiao Q, Werner J, Venkatachalam N, Boonekamp KE, Ebert MP, Zhan T. Cross-Talk between p53 and Wnt Signaling in Cancer. Biomolecules. 2022;12(3). 10.3390/biom12030453 PMC8946298 35327645

[55] Yu J, Zhang L, Hwang PM, Kinzler KW, Vogelstein B. PUMA induces the rapid apoptosis of colorectal cancer cells. Mol Cell. 2001;7(3):673–82. doi: 10.1016/s1097-2765(01)00213-1 11463391

[56] Leszczynska KB, Foskolou IP, Abraham AG, Anbalagan S, Tellier C, Haider S, et al. Hypoxia-induced p53 modulates both apoptosis and radiosensitivity via AKT. J Clin Invest. 2015;125(6):2385–98. doi: 10.1172/JCI80402 25961455 PMC4497762

[57] Salomon D, Sacco PA, Roy SG, Simcha I, Johnson KR, Wheelock MJ, et al. Regulation of beta-catenin levels and localization by overexpression of plakoglobin and inhibition of the ubiquitin-proteasome system. J Cell Biol. 1997;139(5):1325–35. doi: 10.1083/jcb.139.5.1325 9382877 PMC2140206

[58] Lam L, Aktary Z, Bishay M, Werkman C, Kuo CY, Heacock M, et al. Regulation of subcellular distribution and oncogenic potential of nucleophosmin by plakoglobin. Oncogenesis. 2012;1:e4. doi: 10.1038/oncsis.2012.4 23552556 PMC3412635

[59] Miravet S, Piedra J, Miró F, Itarte E, García de Herreros A, Duñach M. The transcriptional factor Tcf-4 contains different binding sites for beta-catenin and plakoglobin. J Biol Chem. 2002;277(3):1884–91. doi: 10.1074/jbc.M110248200 11711551

[60] Fang WK, Liao LD, Gu W, Chen B, Wu ZY, Wu JY, et al. Down-regulated γ-catenin expression is associated with tumor aggressiveness in esophageal cancer. World J Gastroenterol. 2014;20(19):5839–48. 24914344 10.3748/wjg.v20.i19.5839 PMC4024793

[61] Yang L, Chen Y, Cui T, Knösel T, Zhang Q, Albring KF, et al. Desmoplakin acts as a tumor suppressor by inhibition of the Wnt/β-catenin signaling pathway in human lung cancer. Carcinogenesis. 2012;33(10):1863–70. 22791817 10.1093/carcin/bgs226

[62] Jenkinson SR, Barraclough R, West CR, Rudland PS. S100A4 regulates cell motility and invasion in an in vitro model for breast cancer metastasis. Br J Cancer. 2004;90(1):253–62. doi: 10.1038/sj.bjc.6601483 14710237 PMC2395304

[63] Sack U, Walther W, Scudiero D, Selby M, Aumann J, Lemos C, et al. S100A4-induced cell motility and metastasis is restricted by the Wnt/β-catenin pathway inhibitor calcimycin in colon cancer cells. Mol Biol Cell. 2011;22(18):3344–54. 21795396 10.1091/mbc.E10-09-0739 PMC3172260

[64] Wang Z, Burigotto M, Ghetti S, Vaillant F, Tan T, Capaldo BD, et al. Loss-of-Function but Not Gain-of-Function Properties of Mutant TP53 Are Critical for the Proliferation, Survival, and Metastasis of a Broad Range of Cancer Cells. Cancer Discov. 2024;14(2):362–79. doi: 10.1158/2159-8290.CD-23-0402 37877779 PMC10850947

[65] Lo CS, Omar SI, Chang J, Bassey-Archibong B, A Tuszynski J, Jahroudi N, et al. Identification of p53-R175H Q167 and R248 as Residues Most Involved in Its Interaction with Plakoglobin. Re:GEN Open. 2023;3(1):40–51.

