# Supplementary material for: Differential effect of plakoglobin in restoring the tumor suppressor activities of p53-R273H vs. p53-R175H mutants
Source: PLoS One. 2024 Oct 3;19(10):e0306705. doi: 10.1371/journal.pone.0306705 (PMC11449273; doi:10.1371/journal.pone.0306705)
Supplement: S1 Raw images — (PDF) [file pone.0306705.s001.pdf]

Figure 1a - TCE

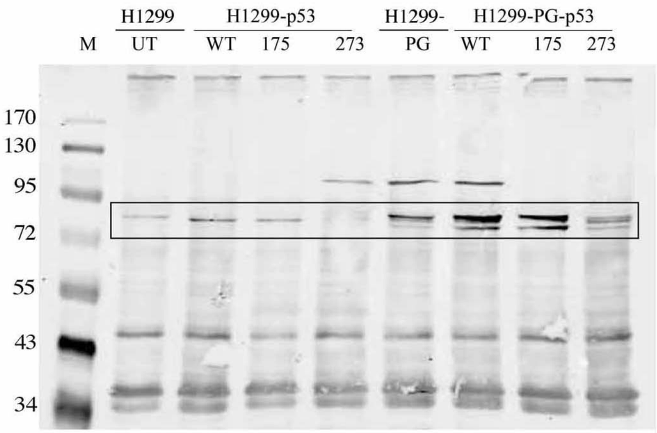

Primary: IB: PG (m) BD 1:1000
Secondary: Goat anti-mouse 800 1:30,000

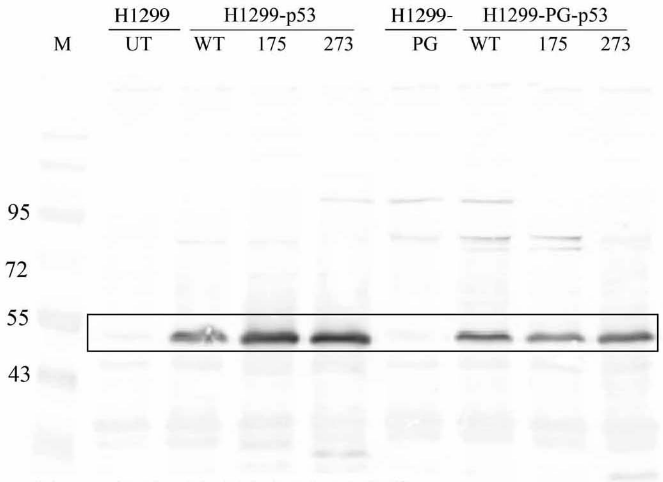

Primary: Reblot IB p53(m) DO-1 1:1000
Secondary: Goat anti-mouse 800 1:30,000

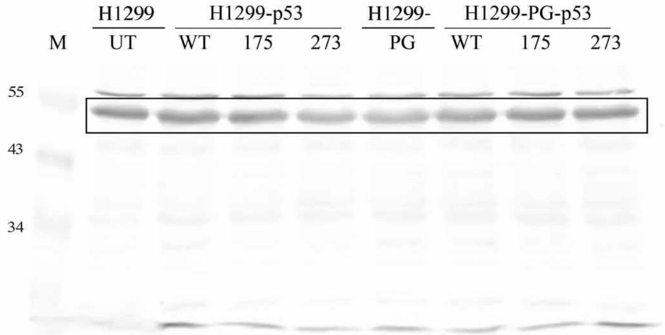

Primary: IB tubulin (E7)
Secondary Goat anti-mouse 1:30,000

Figure 1b

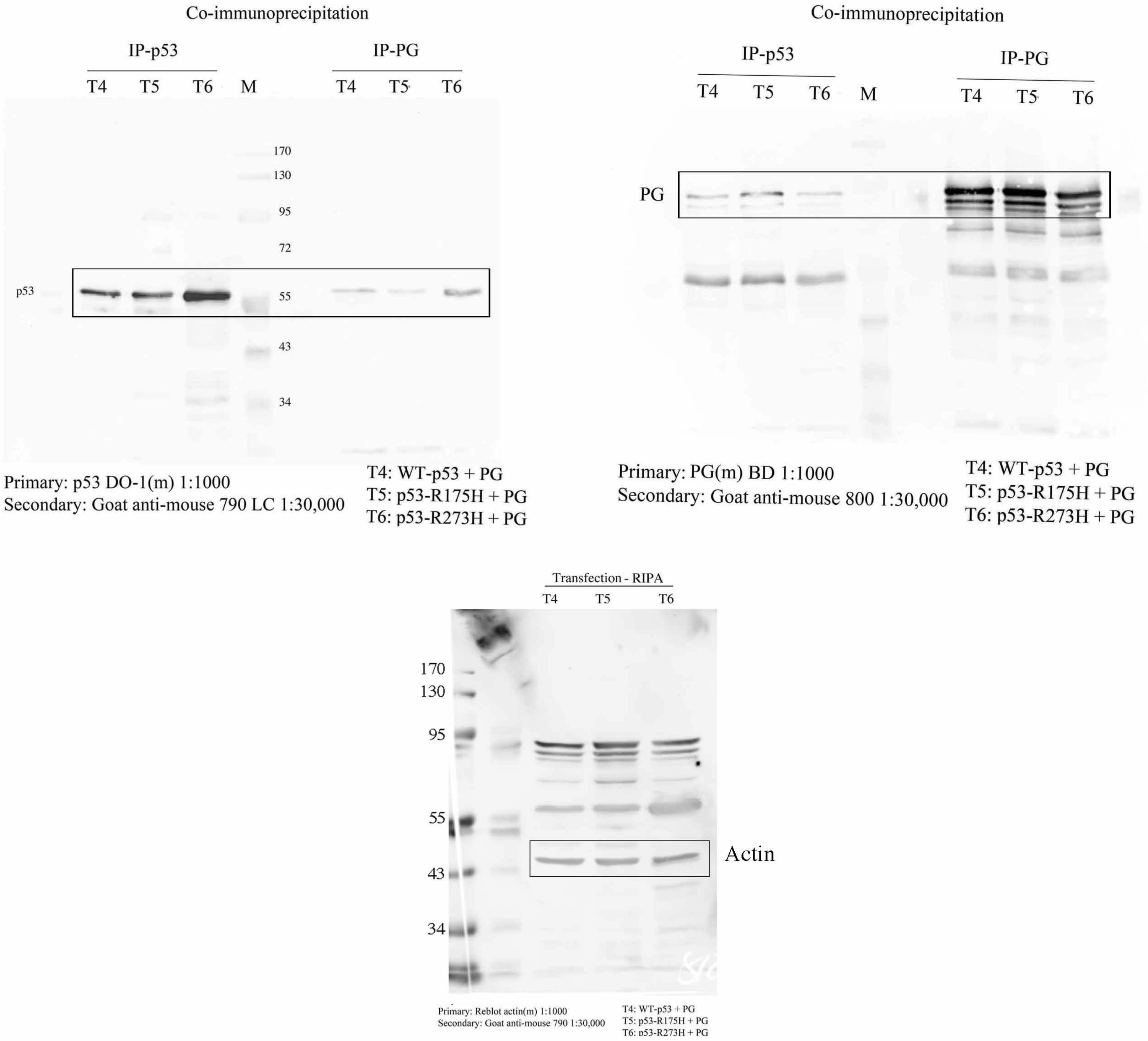
Co-immunoprecipitation
IP-p53
IP-PG
T4 T5 T6 M T4 T5 T6
170
130
95
72
p53
55
43
34
Primary: p53 DO-1(m) 1:1000
Secondary: Goat anti-mouse 790 LC 1:30,000
T4: WT-p53 + PG
T5: p53-R175H + PG
T6: p53-R273H + PG
Co-immunoprecipitation
IP-p53
IP-PG
T4 T5 T6 M T4 T5 T6
PG
Primary: PG(m) BD 1:1000
Secondary: Goat anti-mouse 800 1:30,000
T4: WT-p53 + PG
T5: p53-R175H + PG
T6: p53-R273H + PG
Transfection - RIPA
T4 T5 T6
170
130
95
55
43
34
Actin
Primary: Reblot actin(m) 1:1000
Secondary: Goat anti-mouse 790 1:30,000
T4: WT-p53 + PG
T5: p53-R175H + PG
T6: p53-R273H + PG

Figure 1c

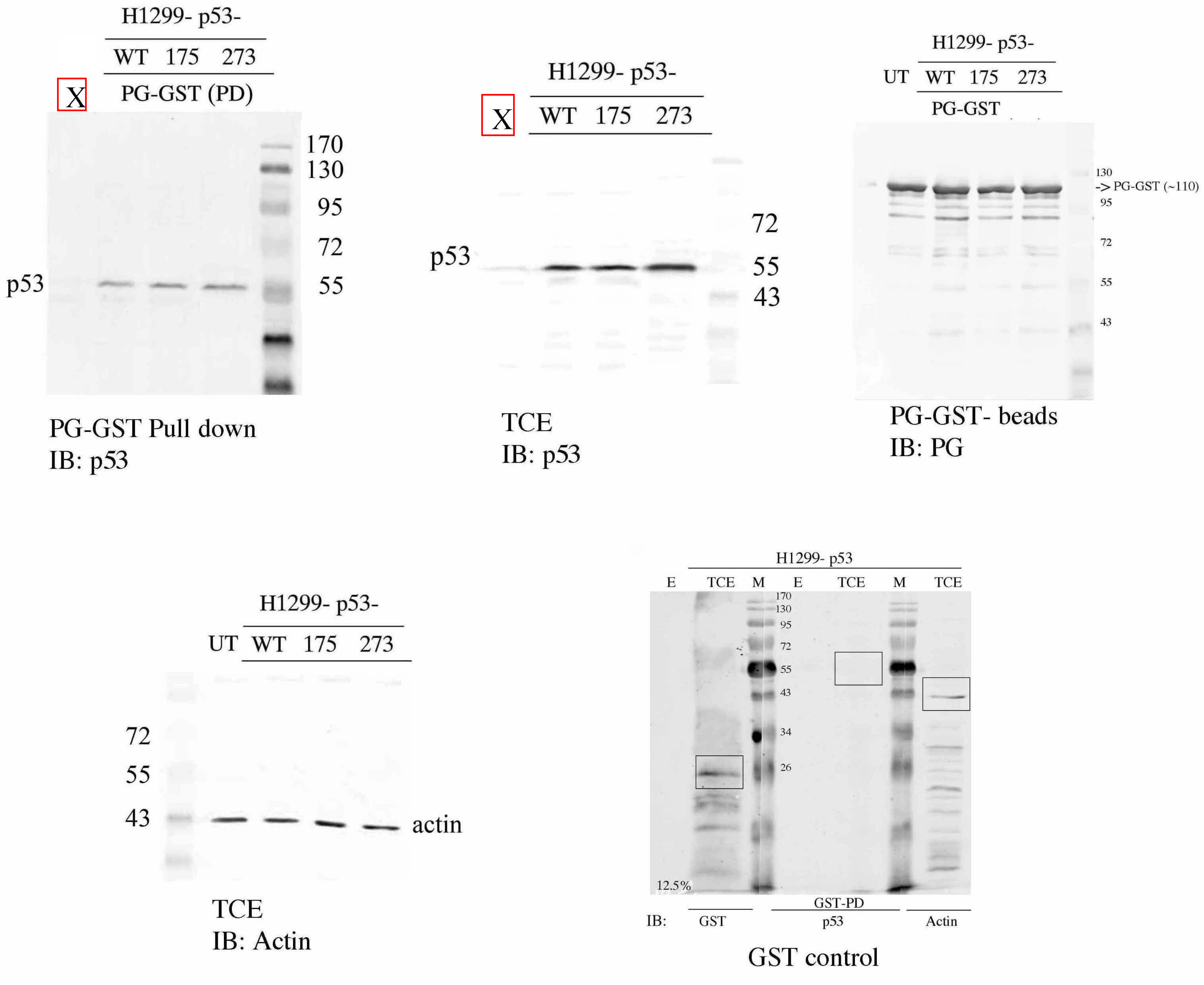
H1299- p53-
WT 175 273
X
PG-GST (PD)
170
130
95
72
55
p53
PG-GST Pull down
IB: p53
H1299- p53-
X
WT 175 273
72
55
43
p53
TCE
IB: p53
H1299- p53-
UT WT 175 273
PG-GST
130
-> PG-GST (~110)
95
72
55
43
PG-GST- beads
IB: PG
H1299- p53-
UT WT 175 273
72
55
43
actin
TCE
IB: Actin
H1299- p53
E TCE M E TCE M TCE
170
130
95
72
55
43
34
26
12.5%
GST-PD
IB: GST p53 Actin
GST control

Figure 1d

Figure 1D- IB: p53

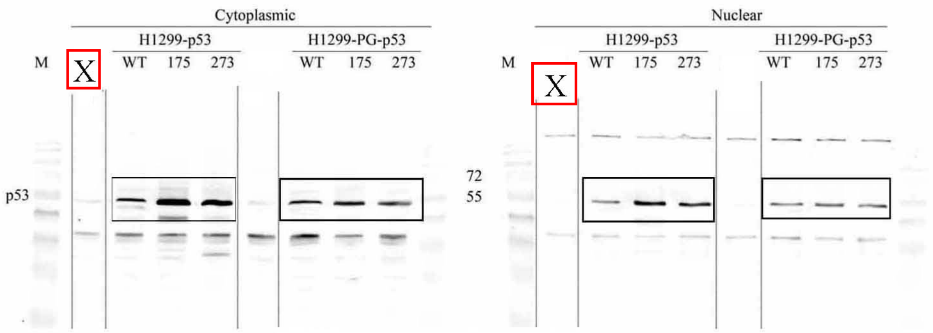

Primary: p53 DO-1(m) 1:1000
Secondary: Goat anti-mouse 800 1:30,000

Figure 1D- IB: PG

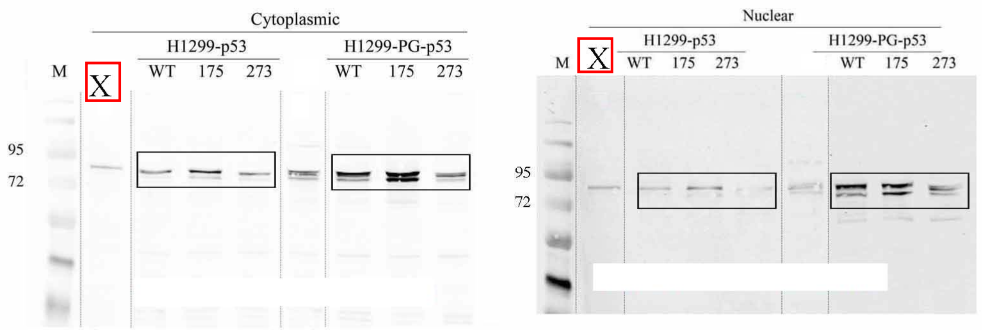

Primary: PG(m) BD 1:1000
Secondary: Goat anti-mouse 800 1:30,000

Primary: PG(m) BD 1:1000
Secondary: Goat anti-mouse 800 1:30,000

Cytoplasmic

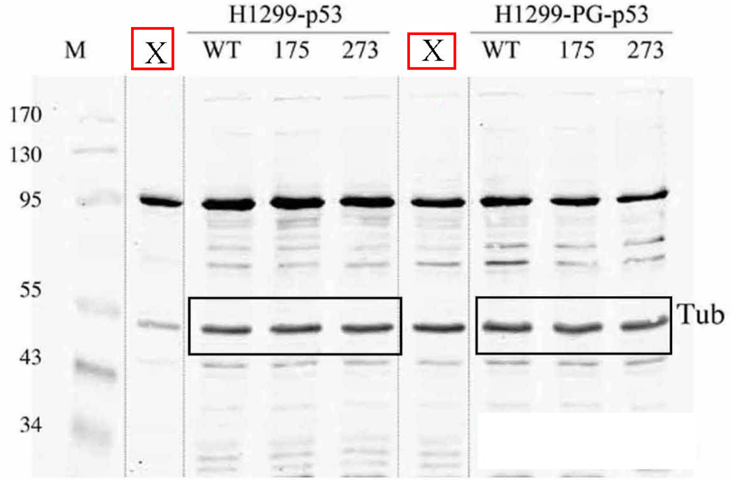

Primary: Reblot Tubulin E7(m) 1:1000
Secondary: Goat anti-mouse 790 LC: 1:20,000

Nuclear

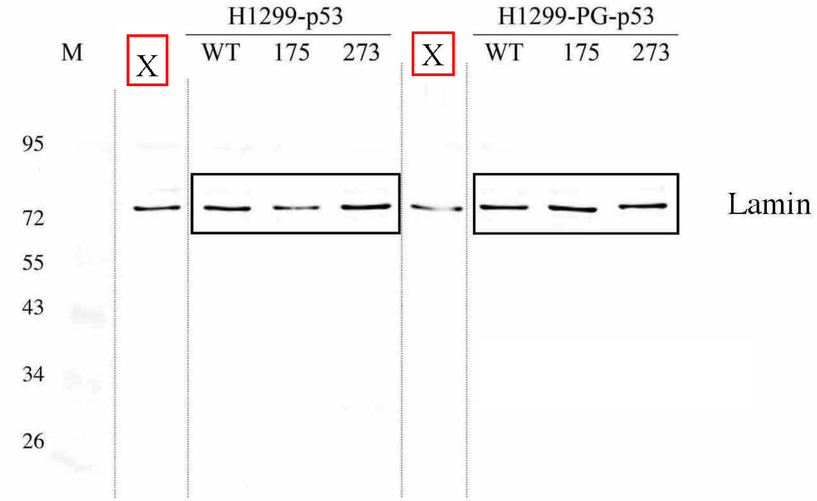

Primary: Reblot lamin (rab) 1:1000
Secondary: mouse anti-rabbit 790 LC 1:30,000

Figure 2a

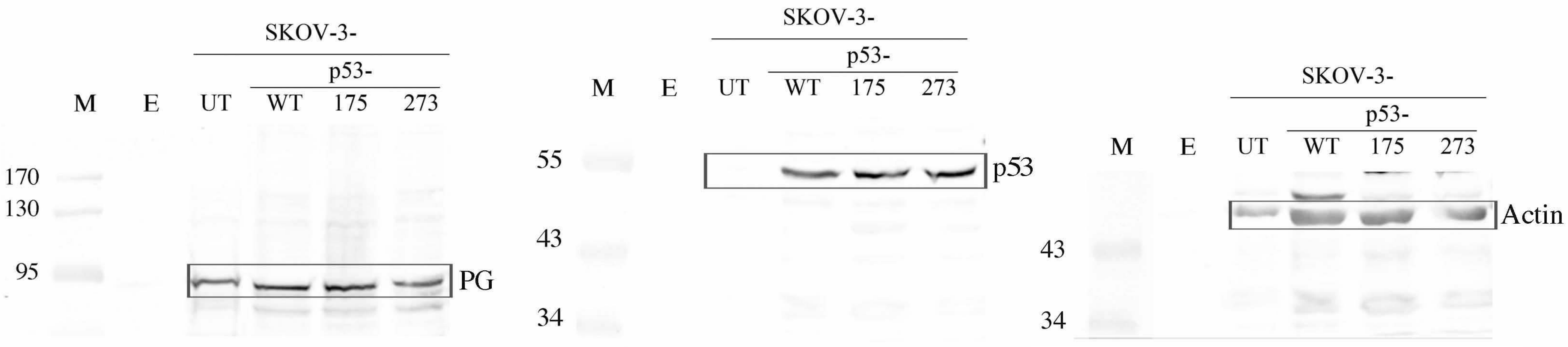

SKOV 3- p53 trnsfection- Above 72 kD
Primary: PG(m) 1:1000
Secondary: Goat anti-mouse 790 LC 1:40,000

SKOV 3- p53 trnsfection- Below 72 kD
Primary: p53(rab) 1:1000
Secondary: Mouse anti-rab 790 LC 1:40,000

SKOV 3-p53 transfection- Below 55 kD
Primary: Actin (m) 1:1000
Secondary: Goat anti-mouse 790 LC 1:40,000

Figure 2b

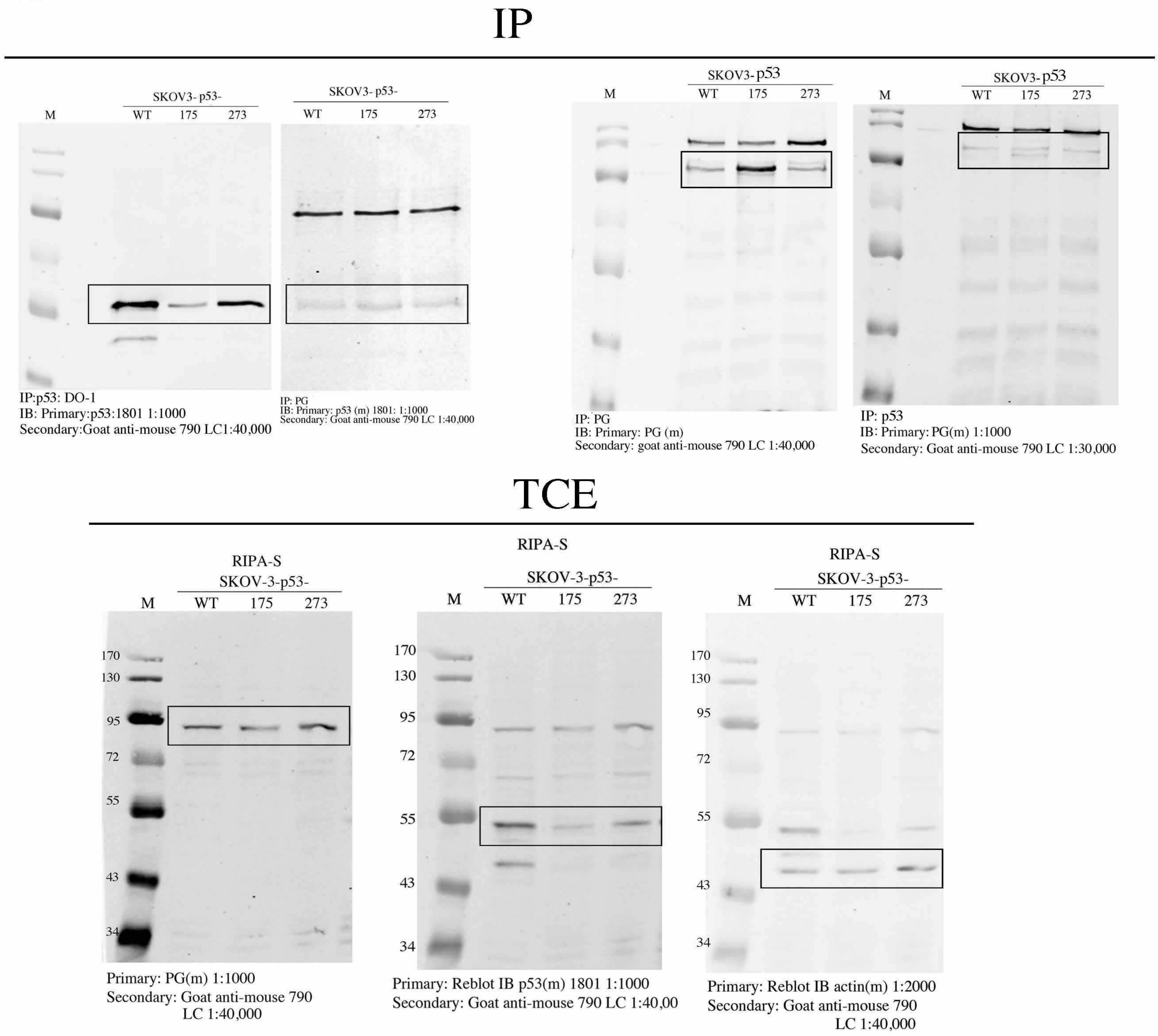

IP
SKOV3- p53-
M WT 175 273
IP:p53: DO-1
IB: Primary:p53:1801 1:1000
Secondary:Goat anti-mouse 790 LC1:40,000
SKOV3- p53-
WT 175 273
IP: PG
IB: Primary: p53 (m) 1801: 1:1000
Secondary: Goat anti-mouse 790 LC 1:40,000
SKOV3- p53
M WT 175 273
IP: PG
IB: Primary: PG (m)
Secondary: goat anti-mouse 790 LC 1:40,000
SKOV3- p53
M WT 175 273
IP: p53
IB: Primary: PG(m) 1:1000
Secondary: Goat anti-mouse 790 LC 1:30,000
TCE
RIPA-S
SKOV-3-p53-
M WT 175 273
170
130
95
72
55
43
34
Primary: PG(m) 1:1000
Secondary: Goat anti-mouse 790
LC 1:40,000
RIPA-S
SKOV-3-p53-
M WT 175 273
170
130
95
72
55
43
34
Primary: Reblot IB p53(m) 1801 1:1000
Secondary: Goat anti-mouse 790 LC 1:40,00
RIPA-S
SKOV-3-p53-
M WT 175 273
170
130
95
72
55
43
34
Primary: Reblot IB actin(m) 1:2000
Secondary: Goat anti-mouse 790
LC 1:40,000

Figure 5

5a

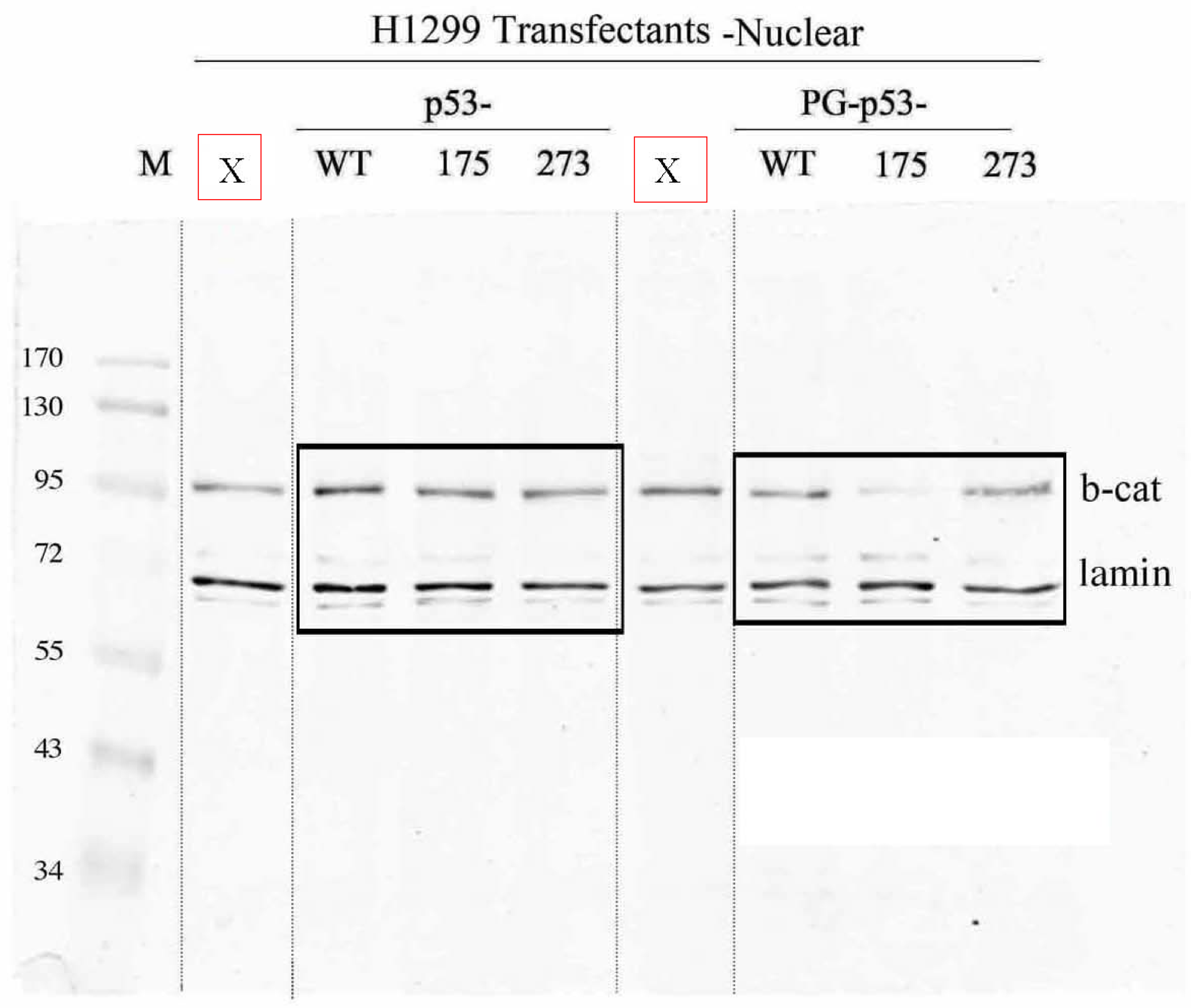

NE-PER- Nuclear
IB: b-cat (m) and lamin (rab)

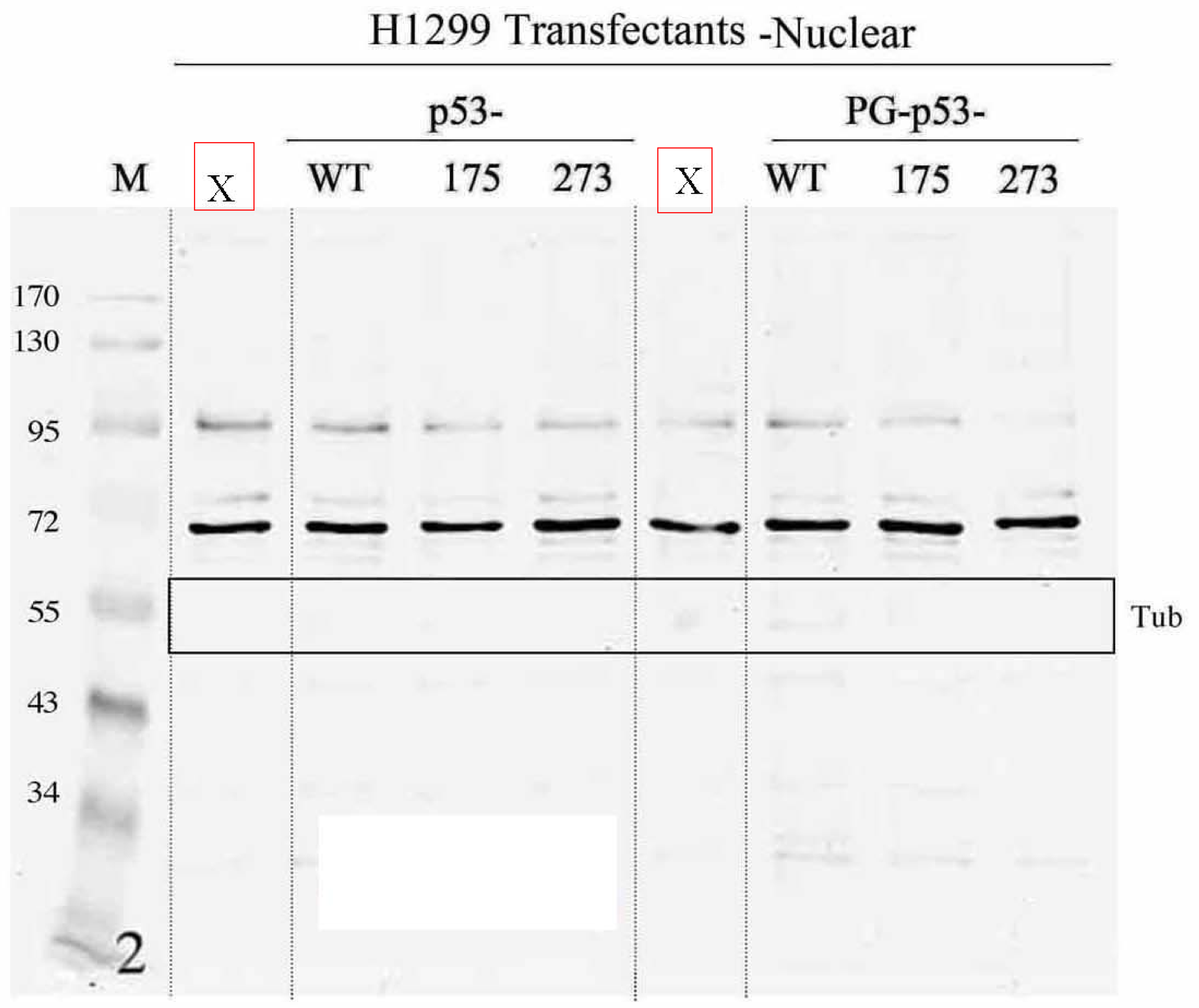

NE-PER- Nuclear
IB: tubulin E7 (m)

5b

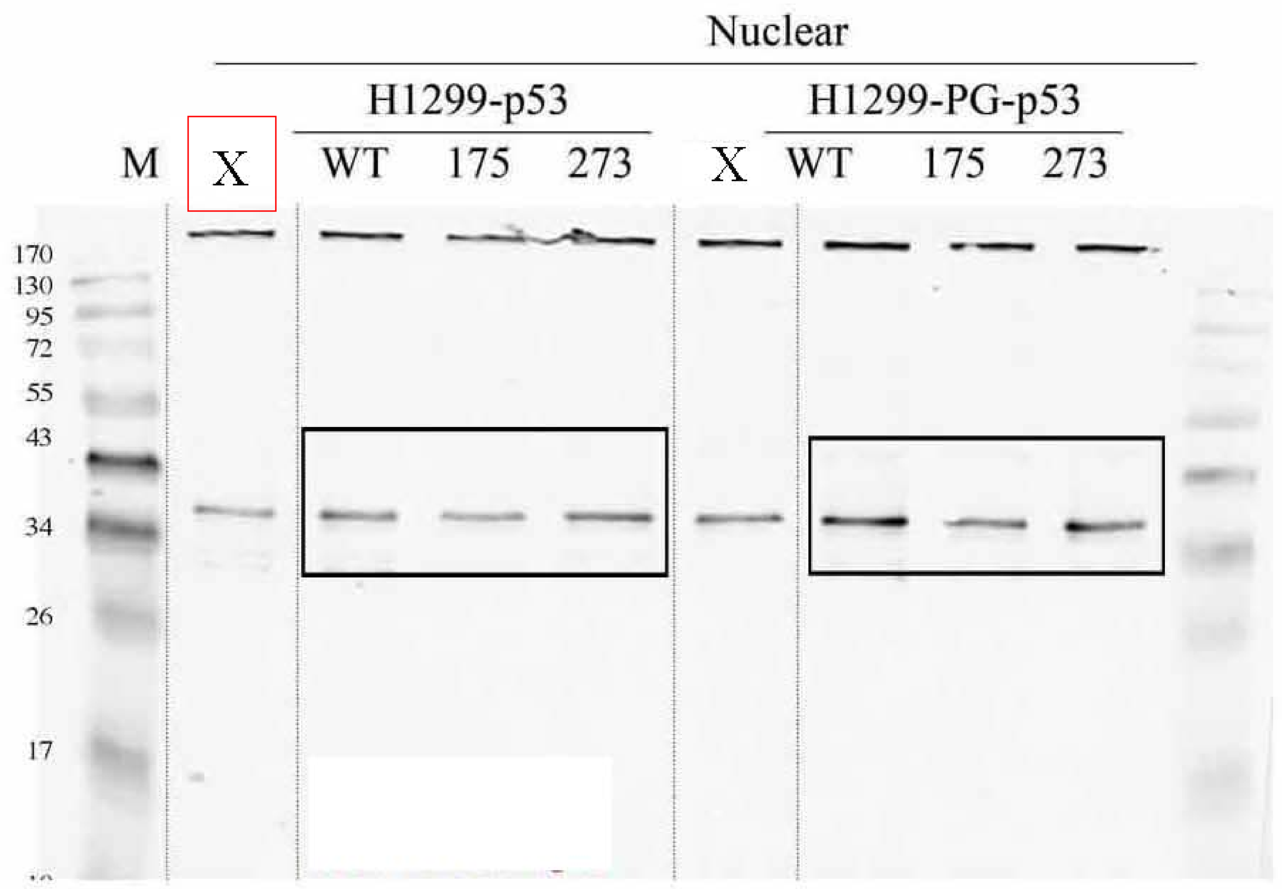

NE-PER- Nuclear
IB: NPM (m)

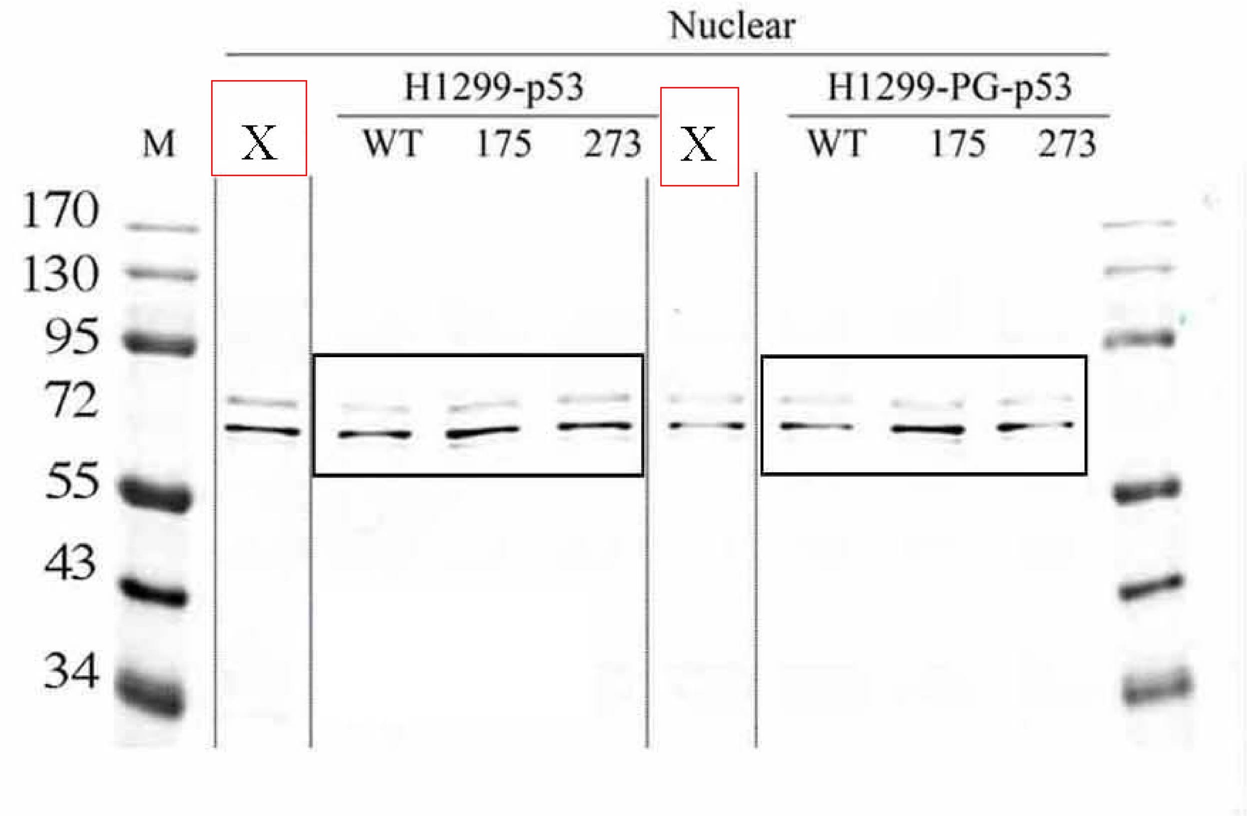

NE-PER- Nuclear
IB: lamin (rab)

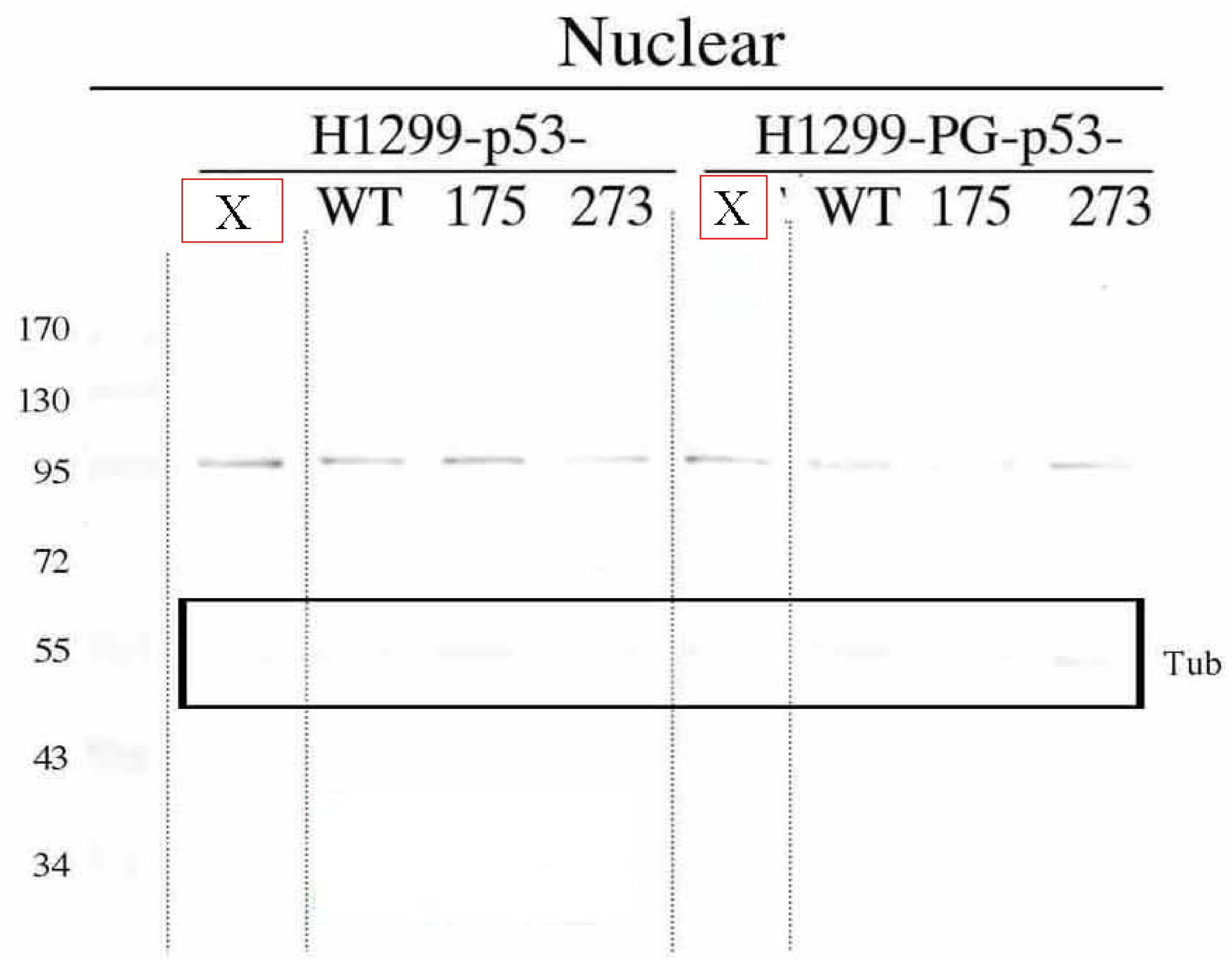

NE-PER- Nuclear
IB: tubulin E7 (m)

Supplementary Figure S1-b

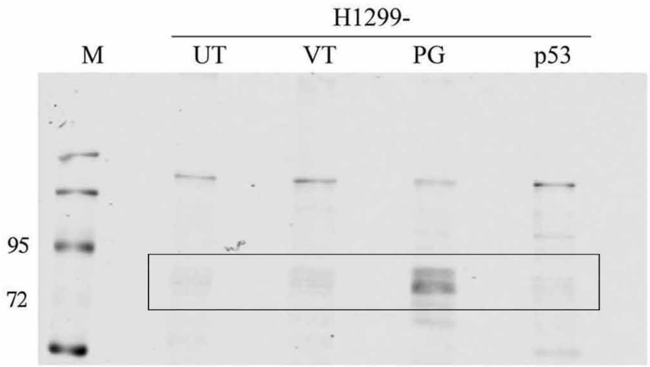

Primary: PG(m) 1:1000
Secondary: Goat anti-mouse 790 LC 1:20,000

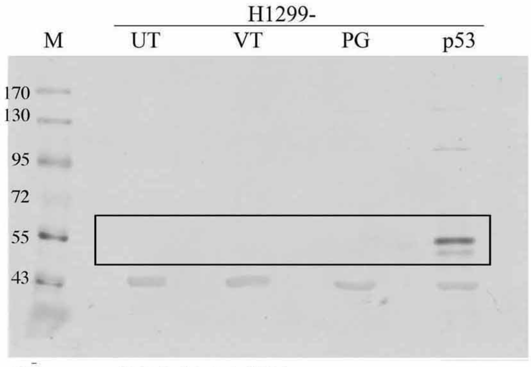

Primary: p53 DO-1(m) 1:1000
Secondary: Goat anti-mouse 790 LC 1:20,000

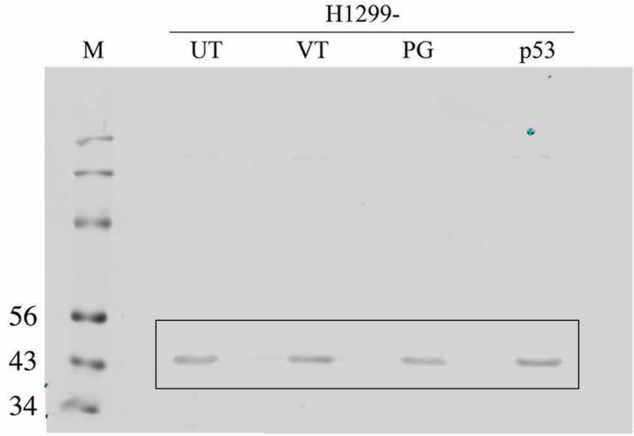

Primary: Actin(m) 1:1000
Secondary: Goat anti-mouse 790 LC 1:20,000
