# Supplementary figures and images for: Differential effect of plakoglobin in restoring the tumor suppressor activities of p53-R273H vs. p53-R175H mutants
Source: PLoS One. 2024 Oct 3;19(10):e0306705. doi: 10.1371/journal.pone.0306705 (PMC11449273; doi:10.1371/journal.pone.0306705)

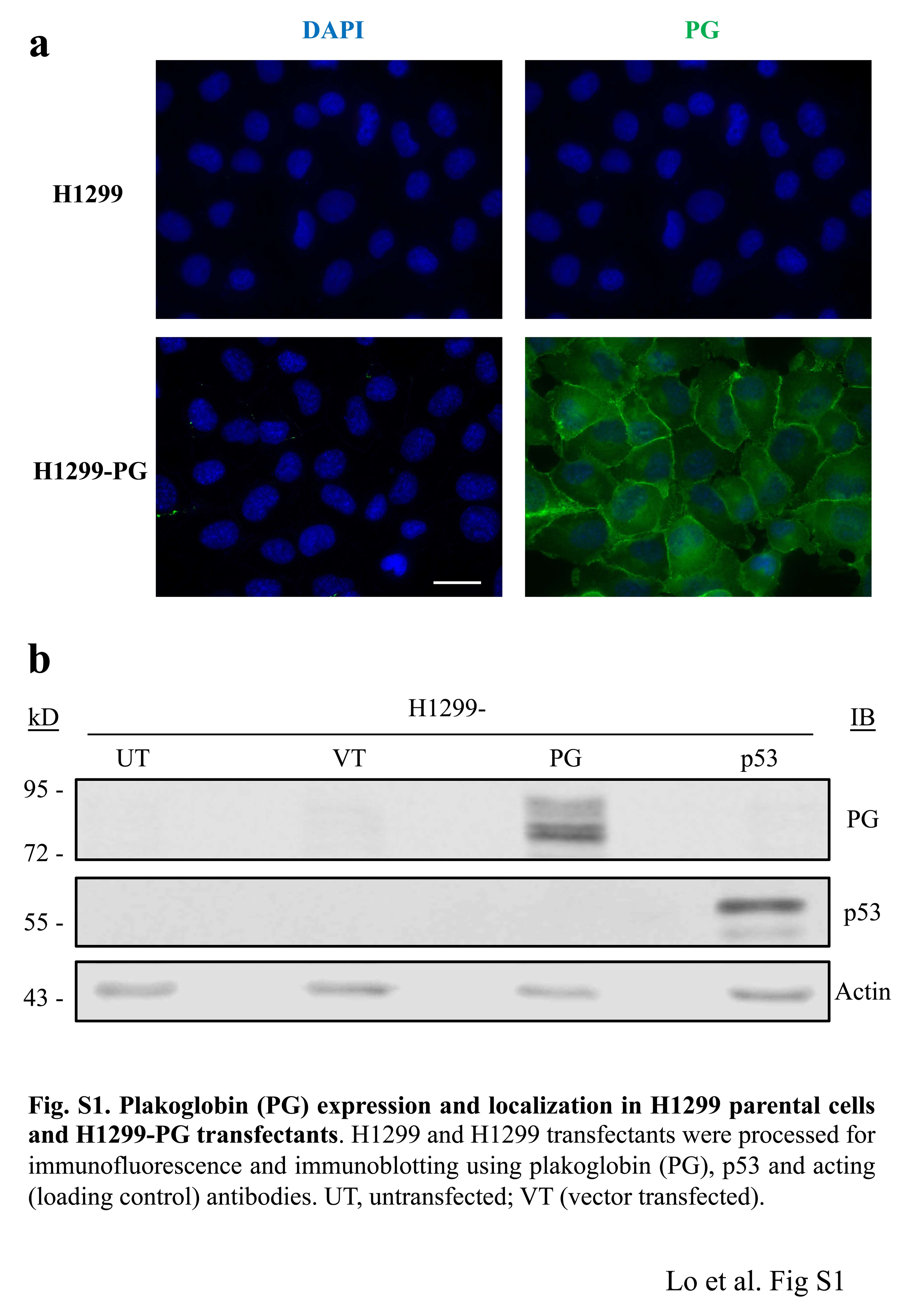

Supplement: S1 Fig — H1299 and H1299 transfectants were processed for immunofluorescence and immunoblotting using plakoglobin (PG), p53 and acting (loading control) antibodies. UT, untransfected; VT (vector transfected). (TIF) [file pone.0306705.s003.tif]

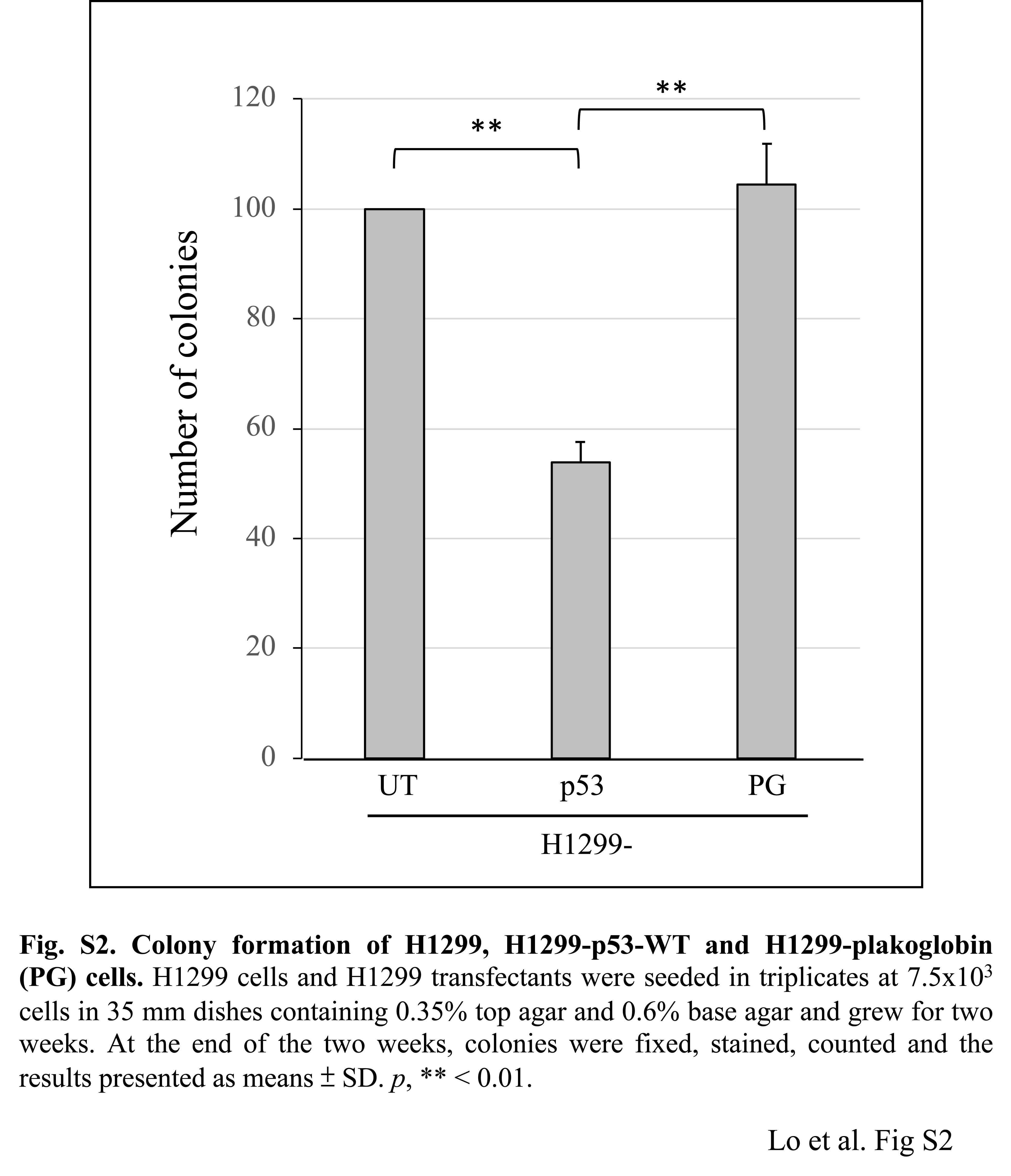

Supplement: S2 Fig — H1299 cells and H1299 transfectants were seeded in triplicates at 7.5x10^3^ cells in 35 mm dishes containing 0.35% top agar and 0.6% base agar and grew for two weeks. At the end of the two weeks, colonies were fixed, stained, counted and the results presented as means ± SD. p, ** < 0.01. (TIF) [file pone.0306705.s004.tif]

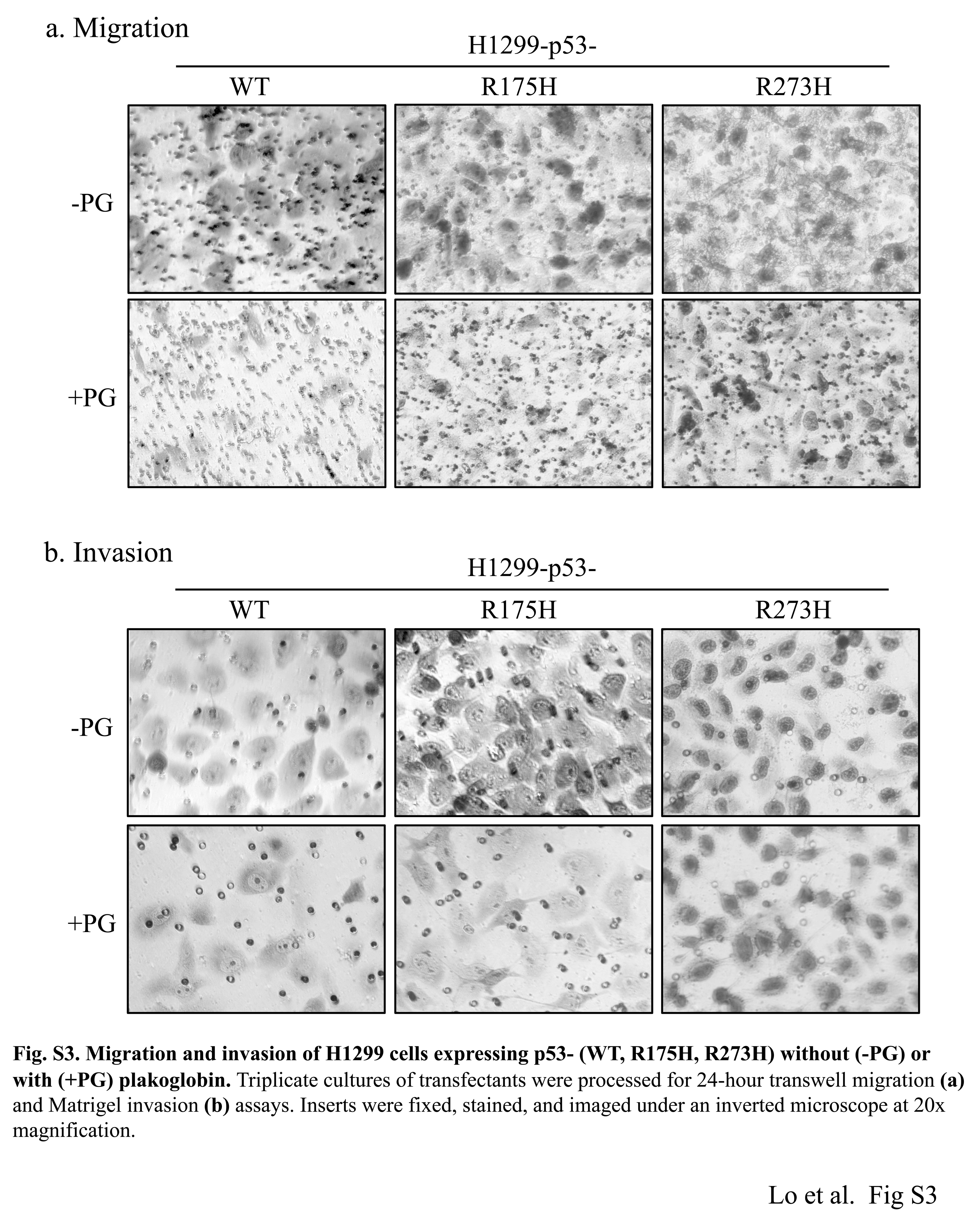

Supplement: S3 Fig — Triplicate cultures of transfectants were processed for 24-hour transwell migration (a) and Matrigel invasion (b) assays. Inserts were fixed, stained, and imaged under an inverted microscope at 20x magnification. (TIF) [file pone.0306705.s005.tif]

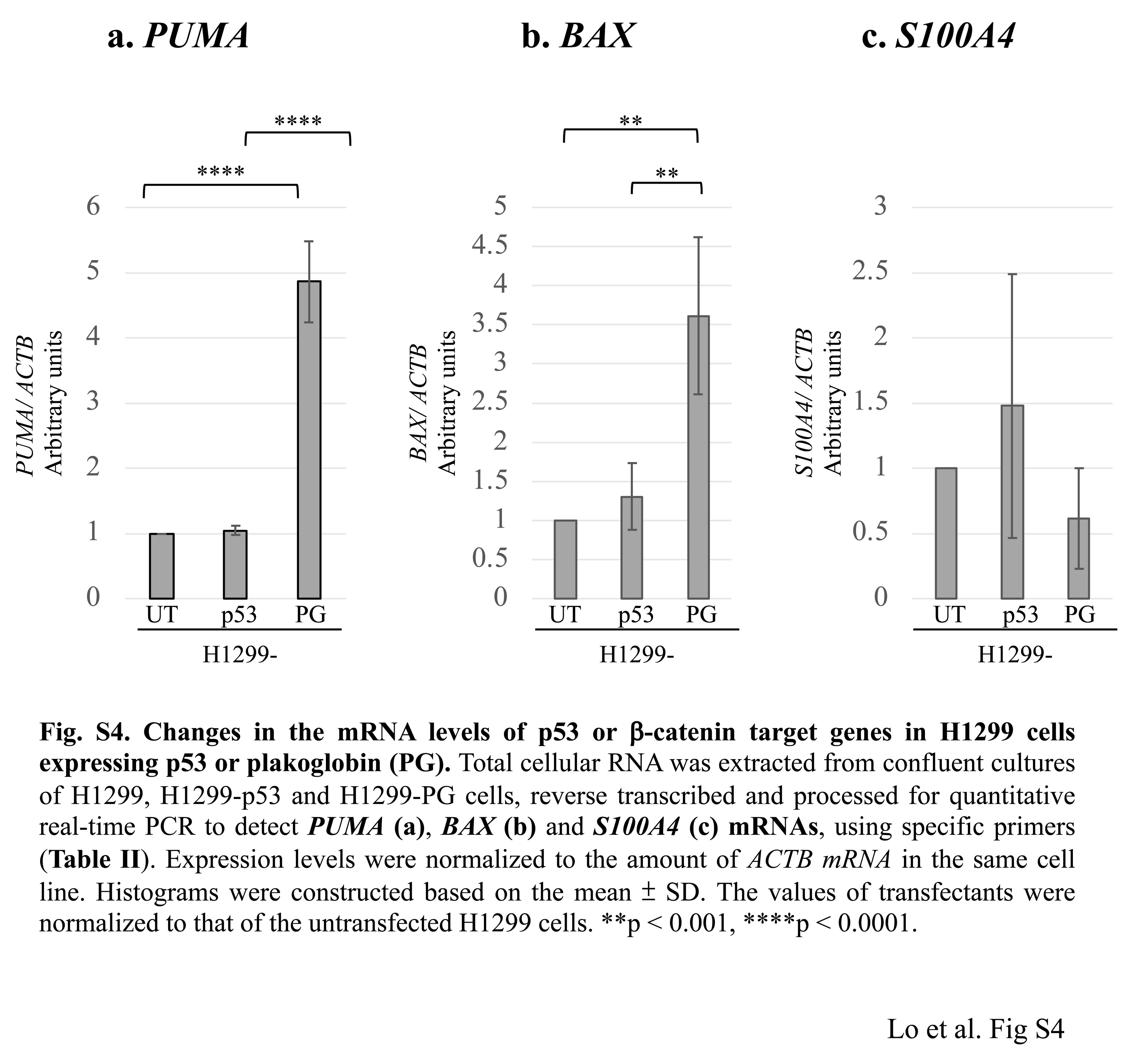

Supplement: S4 Fig — Total cellular RNA was extracted from confluent cultures of H1299, H1299-p53 and H1299-PG cells, reverse transcribed and processed for quantitative real-time PCR to detect PUMA (a), BAX (b) and S100A4 (c) mRNAs, using specific primers (Table 2). Expression levels were normalized to the amount of ACTS mRNA in the same cell line. Histograms were constructed based on the mean ± SD. The values of transfectants were normalized to that of the untransfected H1299 cells. **p < 0.001, ****p < 0.0001. (TIF) [file pone.0306705.s006.tif]

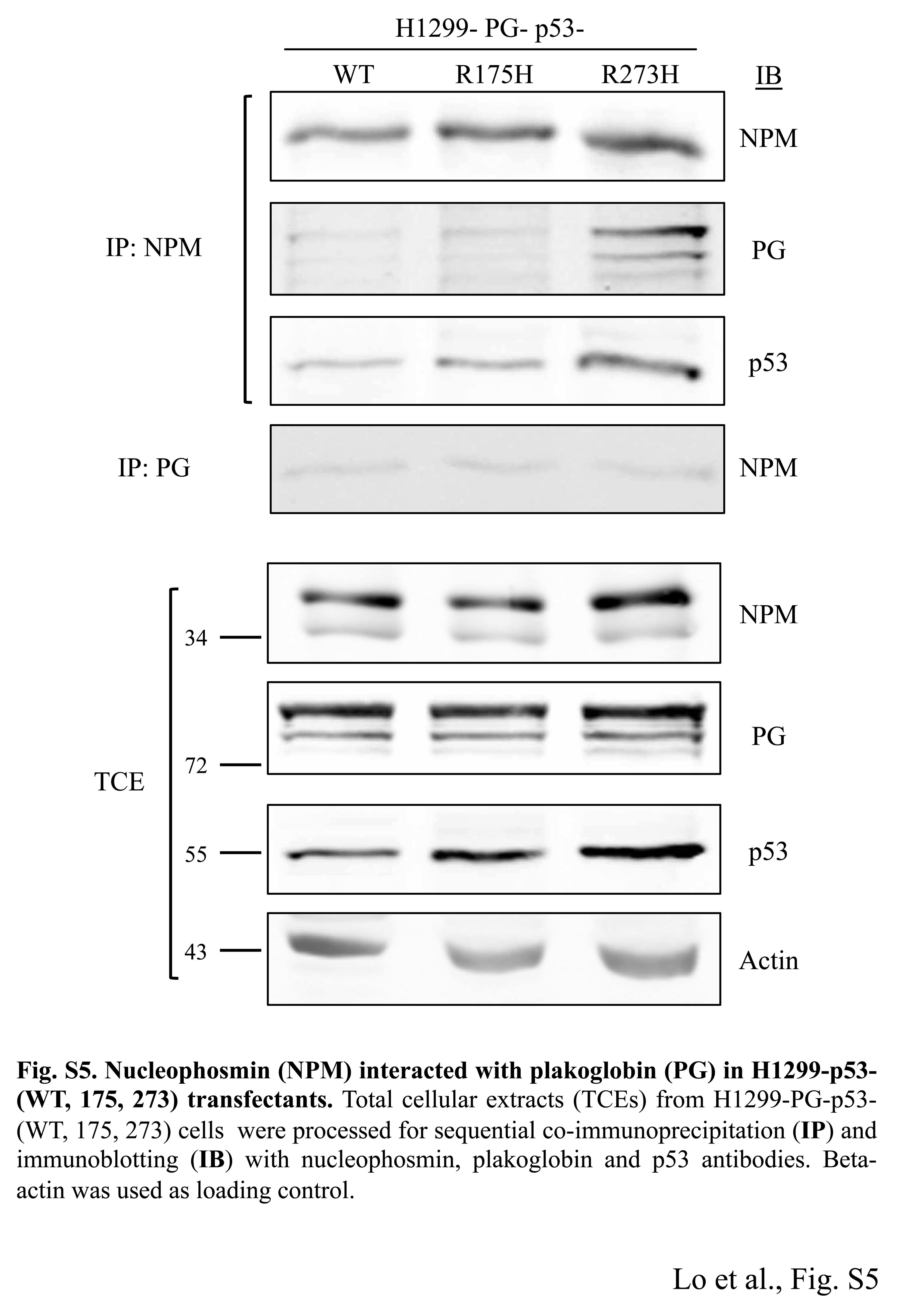

Supplement: S5 Fig — Total cellular extracts (TCEs) from H1299-PG-p53-(WT, 175, 273) cells were processed for sequential co-immunoprecipitation (IP) and immunoblotting (IB) with nucleophosmin, plakoglobin and p53 antibodies. Beta-actin was used as loading control. (TIF) [file pone.0306705.s007.tif]
